# They're Out There, You Know: Sea Turtle Sightings and Strandings in Canadian Pacific Waters

**DOI:** 10.1002/ece3.72513

**Published:** 2026-01-28

**Authors:** Lisa Spaven, Amy Migneault, Karina Dracott, Caitlin Birdsall, Tessa Danelesko, Stephen Raverty, Martin Haulena, John K. B. Ford

**Affiliations:** ^1^ Fisheries and Oceans Canada, Pacific Biological Station Nanaimo British Columbia Canada; ^2^ Ocean Wise Vancouver British Columbia Canada; ^3^ Marine Education and Research Society, Port McNeil British Columbia Canada; ^4^ BC Animal Health Centre Abbotsford British Columbia Canada; ^5^ Vancouver Aquarium Marine Mammal Rescue Society Vancouver British Columbia Canada

**Keywords:** British Columbia, Canada, *Caretta caretta*, *Chelonia mydas*, *Dermochelys coriacea*, *Lepidochelys olivacea*, marine testudine

## Abstract

Pacific sea turtle populations primarily inhabit subtropical and tropical waters, making sightings at the edge of their range in colder high‐latitude regions of the Canadian Pacific particularly uncommon and even rare. This paper presents a comprehensive summary of known occurrences in British Columbia waters from 1931 to 2024, featuring demographics, spatiotemporal distribution, and pathological findings. The dataset contains 247 sea turtle records from four species including 77 previously unpublished records. Leatherback sea turtles ( *Dermochelys coriacea* ) were the most frequently sighted, followed by hard‐shelled sea turtle species: 34 green ( *Chelonia mydas* ), three loggerhead ( *Caretta caretta* ), the first five olive ridley reports ( *Lepidochelys olivacea* ), and 54 unidentified sea turtles. Leatherbacks were primarily observed free‐swimming, whereas almost half of the hard‐shelled sea turtles were found dead or cold‐stunned. This difference may be attributed to the inability of hard‐shelled sea turtles to thermoregulate in high latitude waters. Although leatherback sightings predominantly occurred July through October, hard‐shelled sea turtle records were distributed widely across all months of the year. There were 16 records involving human interactions, of which 75% were attributed to entanglement in fishing gear. Given the rarity of these occurrences and the conservation status of most sea turtle populations, these records provide important insights into high‐latitude habitat use and threats, informing future monitoring and recovery efforts for these at‐risk species.

## Graphical abstract

A comprehensive summary of all known sea turtle occurrences in Canadian Pacific (British Columbia, BC) waters from 1931 to 2024, including demographics, spatiotemporal distribution, and pathologic findings. The dataset contains sightings of 247 sea turtles from four species, including the first five records of olive ridley sea turtles ( *Lepidochelys olivacea* ) in BC waters. Leatherbacks ( *Dermochelys coriacea* ) were the most frequently sighted sea turtle, followed by hard‐shelled sea turtle species: green ( *Chelonia mydas* ), loggerhead ( *Caretta caretta* ), and olive ridley, along with an additional 54 unidentified sea turtles.

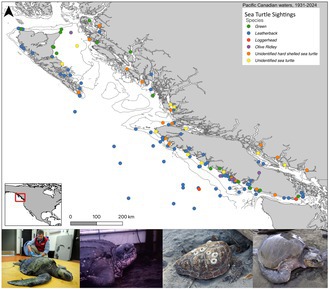

## 1 Introduction

Sea turtles typically inhabit tropical and subtropical waters of the Pacific Ocean, and the temperate waters of western Canada are skirting the range limits for most sea turtle species. Documentation of marine species at the edges of their ranges is critical for effective conservation management and understanding of our changing oceans and climate. Over most of the past century, sea turtles have been regularly reported throughout British Columbia (BC) waters, though infrequently. The first sea turtle sighted in BC was a leatherback in 1931, that was shot and killed for the sheer rarity of its presence. Since then, sporadic records of leatherback sea turtles ( *Dermochelys coriacea* ), green sea turtles ( *Chelonia mydas* ), and loggerhead sea turtles ( *Caretta caretta* ) in BC have been published in Kermode (1932), Carl (1947), Carl (1955), Carl (1960), Radovich (1960), MacAskie and Forrester (1962), Nicholson (1963), Stinson (1984), Hodge and Wing (2000), McAlpine et al. (2002), McAlpine et al. (2004), McAlpine et al. (2007), Spaven et al. (2009), and Halpin et al. (2018). Leatherbacks are the most frequently recorded sea turtle species in Canadian Pacific and adjacent Alaskan and Washington waters (Eisenberg and Frazier 1983; Hodge and Wing 2000; ADFG 2008; Sato 2017), likely due to their thermoregulatory abilities (Bostrom et al. 2010; Casey et al. 2014; Okuyama et al. 2021) which enable high‐latitude foraging on gelatinous prey (James et al. 2006; Bailey et al. 2012; Wallace et al. 2015; Migneault et al. 2023). High‐latitude waters are predominantly outside the usual range for hard‐shelled sea turtles (Wallace et al. 2023), however when found alive in colder waters, hard‐shelled sea turtles are often immobile and in a severe hypothermic state (cold‐stunned), a condition that is most often fatal without human intervention (Schwartz 1978). Abrupt changes in water temperature—due to ocean currents, storms, or wind—can disorient or transport turtles to colder regions (Liu et al. 2019). Green turtles are the most commonly reported hard‐shelled sea turtle in Canadian Pacific and neighboring US waters (Hodge and Wing 2000; Sato 2017; NOAA 2022; K Wilkinson NOAA 2024 unpublished data), though still outnumbered by leatherbacks. Loggerheads have been rarely encountered in coastal waters of the Pacific US Northwest (Hodge and Wing 2000; ADFG 2008; Sato 2017; K Wilkinson NOAA 2024 unpublished data), and there has been only one confirmed and published record in BC (Halpin et al. 2018). Although olive ridley sea turtles ( *Lepidochelys olivacea* ) are the most abundant sea turtle species worldwide (Abreu‐Grobois and Plotkin 2008), sightings of this species off the northwest coast of North America are rare. There are no published records of olive ridleys in BC, though some exist for Washington (Richardson 1999), and Alaska (NOAA 2022; Hodge and Wing 2000).

All three species of sea turtle previously recorded in BC waters are included on the International Union for Conservation of Nature's (IUCN) Red List, as are Olive Ridleys seen in adjacent waters. Based on distribution, sea turtles in BC are likely from the “Endangered” Eastern Pacific green turtle population (Seminoff 2023), the “Least Concern” North Pacific loggerhead turtle subpopulation (Casale and Matsuzawa 2015), and the “Critically Endangered” Western Pacific leatherback population (Benson et al. 2011; Tiwari et al. 2013; Eguchi et al. 2017). The leatherback is the only sea turtle in BC waters that is identified under the Canadian Species At Risk Act (SARA), where it is listed as “Endangered” (COSEWIC 2022). Any Olive Ridleys found off the westcoast of North America are expected to be part of the “Vulnerable” Pacific population (Abreu‐Grobois and Plotkin 2008). Sea turtle populations are negatively impacted by numerous human‐caused threats during all life stages, and throughout their nesting, migratory and foraging habitats (Abreu‐Grobois and Plotkin 2008; Wallace et al. 2013; Casale and Tucker 2017; Seminoff 2023). Pacific Canadian waters represent mainly foraging habitat for leatherbacks, where fisheries entanglement and bycatch are the primary threats (Wallace et al. 2025; COSEWIC 2022). Despite the severe impact from fishing gear and debris entanglement these issues are underreported and understudied (Duncan et al. 2017). Trawl, longline, seine, and gillnet have all been identified as fisheries of concern for sea turtles (Abreu‐Grobois and Plotkin 2008; COSEWIC 2022; Seminoff 2023), in addition to “ghost” or abandoned gear (Duncan et al. 2017). The IUCN Marine Turtle Specialist Group (IUCN MTSC) has evaluated population risks and threats to global sea turtle regional management units (RMU) (Wallace et al. 2025). The most recent assessment found that although overall threat impacts have decreased, fisheries bycatch remains the highest ranked threat across all regions and species, with most “high risk ‐ high threats” RMUs occurring in the Pacific Ocean. Of the sea turtle populations sighted in BC, the Western Pacific leatherback is the only RMU ranked “high risk ‐ high threat” and with critical need for further research. Enhanced monitoring efforts for leatherbacks and identification of critical foraging areas, and threats in the North Pacific are crucial for future management practices in Canadian waters (Gregr et al. 2014; Fisheries and Oceans Canada 2019).

The sporadic nature of published occurrence notes, and the growing number of unpublished reports scattered among organizations, made it challenging to glean an accurate picture of sea turtle presence and distribution in Pacific Canadian waters. This paper provides a summary of all known sea turtle presence and mortality in Canadian Pacific waters, including all previous published records and new occurrences from the past two decades. With climate‐driven shifts in fisheries and species distribution, assessing current knowledge is increasingly important to identify gaps and advance conservation efforts, especially at the limits of sea turtle range.

## 2 Methods

### 2.1 Data Sources

The Canadian Pacific sea turtle dataset includes records of alive and dead turtles acquired through historical investigations, publications and reports, outreach and citizen science efforts, rehabilitation and postmortem examinations, questionnaires, and at‐sea surveys. Targeted sea turtle aerial surveys were conducted in 2005, 2006, and 2007 (Spaven et al. 2009). Since 2002, DFO's cetacean aerial and vessel surveys (Spaven et al. 2009; Ford et al. 2010) have included sea turtles as secondary target species. Adding to the initial efforts of Spaven et al. 2009, at‐sea observer reports, commercial fishery logbooks and bycatch data were queried for reference to turtles.

Questionnaires were sent out in 2003 (Spaven et al. 2009) and again in 2014 to solicit reports of sea turtle encounters in BC waters (*n* = 1478 and 1663 in 2003 and 2014 respectively). Questionnaires were sent to active BC commercial fishing license holders, and a variety of marine‐based operators, organizations, and governments including First Nations (see Appendix A for example questionnaire). Respondents were asked to provide details of their sea turtle sightings (e.g., location, date, physical turtle description, animal behavior and interaction details) and were given visual species identification guides for further verification.

Opportunistic sightings reports were solicited through websites and public outreach presentations by the Marine Education and Research Society (MERS), DFO, and the Ocean Wise Sightings Network (OWSN) (formerly known as the BC Cetacean Sightings Network (BCCSN)). Printed leatherback awareness materials (Appendix B1) and an animated video (Appendix B2) were also shared. Reporting options included toll‐free telephone hotlines, email, online forms, logbooks, and the WhaleReport app (Scott et al. 2024; Appendix B3).

### 2.2 Sea Turtle Stranding Response

Where possible, collected sea turtles were admitted to the Vancouver Aquarium Marine Mammal Rescue Society (VAMMR) for rehabilitation and/or presented to the BC Animal Health Centre (AHC) for postmortem examination. Assessment and stabilization of cold‐stunned sea turtles included a physical exam and diagnostics (i.e., bloodwork, body temperature, electrocardiography, ultrasound, and radiography). Turtles were gradually warmed at 1°C–2°C per day to reach species‐specific environmental temperatures. Supportive care included intracoelomic fluids, prophylactic antibiotics, and other medications as needed. Once active, turtles were reintroduced to water for increasing durations and continued warming.

Necropsies followed standard protocols. Representative tissues were collected and preserved in 10% neutral buffered formalin for histopathology, and fresh samples submitted for routine microbiology and, in select cases, attempted fungal isolation. Fixed tissues were processed through graded alcohols and xylene, embedded in paraffin, sectioned to 5 μm, and stained with hematoxylin and eosin by an automated processor. Slides were reviewed by a board‐certified veterinary pathologist, and lesions were identified and scored.

### 2.3 Species, Spatial and Temporal Quality Control

As per Spaven et al. (2009), a turtle report was considered a duplicate (i.e., not distinct) if it occurred within 24 h of another report within a 5 km radius, unless both reports were by the same observer moving along an unchanged heading. Records were confirmed to species if the identification was certain or highly probable based on the description of the carapace shape and color, the animal's size, overall behavior, and photos/videos. Photographic evidence was forwarded to international experts for species confirmation where necessary. All other records were classified as either “unidentified hard‐shelled sea turtle,” or “unidentified sea turtle” using the best available information.

Age class and sex determinations were confirmed by veterinarian or pathologist examination. Any sea turtle morphometric measurements were classified as either “measured” curved carapace length (CCL), straight carapace length (SCL), straight carapace width and weight resulting from an examination, or simply “estimated” SCL from visual observation (noted by ~ in tables).

Location coordinates were georeferenced from details provided. Records with insufficient location accuracy (> 5 km radius) were not mapped. Coordinates were assigned to confidence categories (Exact, Approximate, Best Guess) used to inform inclusion in spatial and temporal analyses. “Approximate” was assigned where the provided description was within 5km accuracy of where the sighting likely occurred, while “Best Guess” indicates poor information. Some record coordinates were amended to better reflect location accuracy based on observer descriptions, even if previously published. Records were categorized into three latitudinal BC sub‐regions from the coast out to the Canadian Exclusive Economic Zone (EEZ): (1) Southern BC ‐ South of Cape Scott encompassing all of Vancouver Island and inshore waters to the Washington State border, (2) Central BC ‐ between Cape St James and Cape Scott encompassing all of Queen Charlotte Sound and the central mainland coast of BC, and (3) Northern BC ‐ North of Cape St James encompassing all of Haida Gwaii, the northern portion of the BC mainland coast to the Alaskan border. Where possible, temporal information was categorized into seasons in alignment with Spaven et al. (2009): (1) Winter—January through March, (2) Spring—April to June, (3) Summer—July to September, and (4) Fall—October to December. Figures were created using R Studio version 2024.04.02 (Posit Team 2025) and R version 4.4.1 (R Core Team 2024) and QGIS (2.16.3).

### 2.4 Assigning Sea Surface Temperature

Average monthly sea surface temperature (SST) records were extracted from the Environmental Research Division, Southwest Fisheries Science Centre, National Oceanic and Atmospheric Administration, ERDAPP data search engine software using the dataset titled “HadISST Average Sea Surface Temperature, 1°, Global, Monthly, 1870‐present” (ERDDAP—HadISST Average Sea Surface Temperature (SST), 1°, Global, Monthly, 1870–present—Data Access Form). Records of live, free‐swimming sea turtles with data that included both an exact year and month with exact or approximate coordinates (reports indicating the sighting occurred within a 5‐km radius), were matched to the monthly average SST data provided by the ERDAPP search engine.

## 3 Results

**FIGURE 1:**
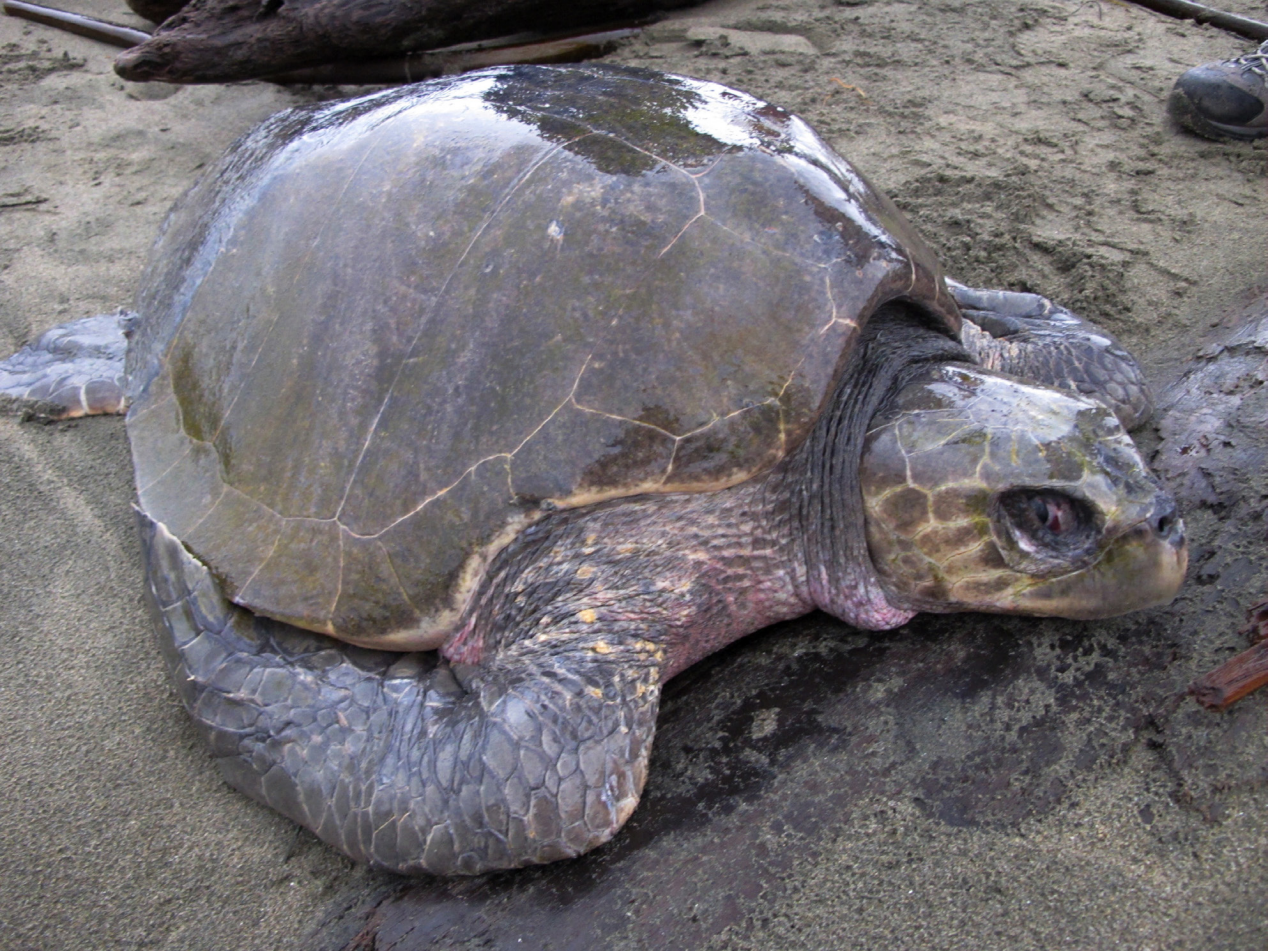
Olive ridley sea turtle found on Wickaninnish Beach within Pacific Rim National Park Reserve on November 22, 2011 (photo credit: Parks Canada).

Here we add 78 previously unpublished sea turtle records in Canadian Pacific waters from 1960 to 2024. This includes 32 distinct leatherback turtles, 14 greens, the first reports of olive ridleys (*n* = 5) (Figure 1), two loggerheads, 15 unidentified hard‐shelled sea turtles, and 10 unidentified sea turtles. Many of these unpublished hard‐shelled sea turtle records originated from the initial historical investigations and the 2003 questionnaire from Spaven et al. (2009) but these records were not published as they were not the focus of that effort. In total 201 and 203 responses were received to the 2003 and 2014 questionnaires respectively, yielding 81 sea turtle records, 23 of which are previously unpublished. Outreach efforts resulted in 52 new records, and 2 new sightings were opportunistically obtained from cetacean‐focused vessel surveys. A live, free‐swimming leatherback was observed during a DFO cetacean vessel survey in July 2016 (LB139), as well as an unidentified hard‐shelled sea turtle in August 2017 (UHS18). Commercial fishers and fishing guides contributed 39% of new reports during fishing operations or in transit, while government researchers and recreational boaters accounted for 13% each. No references to turtles were found in any DFO commercial fishery catch reports or logbooks, and dedicated aerial surveys did not yield any sightings.

There are now 248 distinct sea turtles on record from four different species in Canadian Pacific waters, spanning 1931–2024 (Table 1). Details of all sea turtle records can be found in Table 2. Note that some records include more than one turtle and thus numbers hereafter refer to the total number of turtles rather than the number of reports. Confirmed leatherback records total 151 turtles since 1931. Greens total 34 turtles since 1954. This dataset also contains five olive ridleys, three loggerheads, 20 hard‐shelled sea turtles not identified to species, and 35 records unidentified beyond the Superfamily Chelonioidea (sea turtle). On average there have been 2.8 sea turtles reported in BC per year since 1931.

**TABLE 1:** Summary of all sea turtle records from Pacific Canadian waters, 1931–2024, including total numbers alive and dead, rehabilitated and post mortem exams, as well as straight carapace length ranges, age class, and sex by species.

Sea turtle species	New data	All data	Alive at time of report	Dead	Estimated SCL range	Sex	Age class	Response
Leatherback	32	151	138	13	100–300 cm (*n* = 55)			1 necropsy
Green	14	34	15	19	32–102 cm (*n* = 19)	6 male 1 female	5 adult 2 subadult 1 juvenile	4 rehab 6 necropsy
Olive Ridley	5	5	3	2	55–90 cm (*n* = 4)	3 male 1 female	3 adult 1 subadult	2 rehab 3 necropsy
Loggerhead	2	3	2	1	63–66 cm (*n* = 2)	1 female	1 subadult	1 rehab
Unidentified hard shelled	15	20	16	4	75–125 cm (*n* = 6)			
Unidentified	10	35	32	3	180–250 cm (*n* = 2)			
Total	78	248	206	42		9 male 3 female	8 adults 4 subadult 1 juvenile	7 rehab 10 necropsy

**TABLE 2:** All known sea turtle records in Pacific Canadian waters, 1931 to 2024, including species confidence, animal condition, dates, locations, morphometric measurements, sea surface temperature, and published data sources.

Record #	Species	ID confidence	Event type	Animal condition	Date	Region	Sighting location	Latitude	Longitude	Coordinate confidence	Sea surface temp. (C)	Length (cm)	Width (cm)	Comment	Data source
**Leatherback sea turtle**															
LB1	Leatherback	Certain	Shot	Dead, fresh	16 Aug 1931	Southern BC	Bajo Reef—8 NM S of	49.450	−126.883	Exact		290	300	First ever leatherback turtle reported in BC waters. Shot dead. Photos. Lengths may include head and tail. Carapace collected and donated to Royal BC Museum (RBCM # 460)	Kermode (1932)
LB2	Leatherback	Certain	Shot	Dead, fresh	30 Aug 1931	Southern BC	Bajo Reef—8 NM S of	49.460	−126.850	Exact		215		Observed 2 weeks after the first turtle at similar location. Shot dead. Both turtles were similar in size and weight. Lengths may include head and tail	Kermode (1932)
LB3	Leatherback	Certain	Shot	Dead, fresh	Summer 1934	Southern BC	Nootka Island	49.633	−126.917	Best guess		137	320	Possibly same turtle as first 1931 turtle although no measurements or weights match. Shot dead	Nicholson (1963)
LB4	Leatherback	Certain	Sighting	Alive	14 Mar 1947	Southern BC	Denman Island	49.580	−124.750	Best guess		~120		Observed on at least two occasions but recorded as single sighting as very likely resightings of the same animal	Carl (1947)
LB5	Leatherback	Certain	Sighting	Alive	16 Sep 1947	Southern BC	Cordova Bay, Saanich—200 yds off Sayward Beach	48.550	−123.350	Exact	12.87	~120			Carl (1960)
LB6	Leatherback	Certain	Sighting	Alive	20 Sep 1954	Southern BC	LaPerouse Bank	48.700	−125.620	Best guess				Might be same as 21 Sep 1954 turtle at Pachena but reported seperately by MacAskie and Forrester so reported both here too	MacAskie and Forrester (1962)
LB7	Leatherback	Certain	Sighting	Alive	21 Sep 1954	Southern BC	Esperanza Inlet	49.800	−127.200	Approximate	14.47				MacAskie and Forrester (1962)
LB8	Leatherback	Certain	Sighting	Alive	21 Sep 1954	Southern BC	Pachena Point—2 NM W of	48.718	−125.146	Exact	15.52			Might be same as 20 Sep 1954 turtle at LaPerouse Bank, but reported seperately by MacAskie and Forrester so reported both here too	MacAskie and Forrester (1962)
LB9	Leatherback	Certain	Sighting	Alive	23 Sep 1954	Southern BC	Brooks Bay, Brooks Peninsula	50.167	−127.967	Approximate	13.53			2 free swimming turtles. Not apparent if turtles were a pair or separate. Might be the same as Esperanza sighting few days earlier but impossible to know	MacAskie and Forrester (1962)
LB10	Leatherback	Certain	Entanglement	Alive, died	14 Jul 1957	Southern BC	Useless Inlet, Barkley Sound	48.967	−125.100	Approximate	14.98	180	86	Entangled in commerical gillnet and later died. No necropsy or further assessment	Radovich (1960)
LB11	Leatherback	Certain	Sighting	Alive	Aug 1958	Central BC	Goose Bank, Queen Charlotte Sound	51.583	−129.000	Best guess		~250		Feeding on fish guts off stern of troller	Spaven et al. (2009, 2003); questionnaire
LB12	Leatherback	Certain	Sighting	Alive	1 Nov 1959	Southern BC	McNeill Bay—near Trial Island	48.400	−123.300	Exact	10.4	~240	~150	Coordinates corrected from Stinson 1984	Carl (1960)
LB13	Leatherback	Certain	Sighting	Alive	23 Sep 1961	Northern BC	Sedgwick Bay	52.600	−131.533	Exact	13.99	180		Photos	MacAskie and Forrester (1962)
LB14	Leatherback	Certain	Sighting	Alive	Mid Jul 1970	Central BC	Rivers Inlet—near Sharbau Island	51.417	−127.750	Approximate	12.33			Coordinates corrected from Stinson 1984	Stinson (1984)
LB15	Leatherback	Certain	Sighting	Alive	Around 1972	Southern BC	Cape Cook—S of	49.917	−127.833	Best guess					Spaven et al. (2009, 2003); questionnaire
LB16	Leatherback	Certain	Sighting	Alive	Aug 1975	Southern BC	Barkley Canyon	48.350	−125.967	Best guess					Spaven et al. (2009, 2003); questionnaire
LB17	Leatherback	Certain	Sighting	Alive	Aug 1976	Southern BC	Ucluelet Harbour—24 NM S of	48.500	−125.500	Best guess		~250			Spaven et al. (2009, 2003); questionnaire
LB18	Leatherback	Probable	Sighting	Alive	Summer 1977	Southern BC	Swiftsure Bank	48.533	−124.983	Best guess				Observed while trolling for salmon	Spaven et al. (2009); hotline/outreach
LB19	Leatherback	Certain	Sighting	Alive	14 Sep 1977	Southern BC	Ucluth Peninsula—between Big Bank and Ucluelet Harbour	48.920	−125.630	Approximate	15.65			RBCM #695	Stinson (1984)
LB20	Leatherback	Certain	Sighting	Alive	Jun 1980	Southern BC	Ucluelet Harbour—5 NM W of Amphitrite Lightstation	48.922	−125.667	Exact	12.25				Spaven et al. (2009, 2003); questionnaire
LB21	Leatherback	Probable	Entanglement	Alive	Late Jul or early Aug 1980	Central BC	Cape Mark—close to beach	52.117	−128.550	Exact	13.91			Released alive from commercial seine net	Spaven et al. (2009); hotline/outreach
LB22	Leatherback	Certain	Sighting	Alive	Around Aug 1980	Southern BC	La Perouse Bank	48.750	−125.833	Best guess				Observed while trolling for salmon	Spaven et al. (2009, 2003); questionnaire
LB23	Leatherback	Probable	Sighting	Alive	Late 1960s to early 1980s	Southern BC	Vancouver Island—40‐170 NM offshore	CBD	CBD	Uncertain				Observed up to 6 turtles in a day sometime during fisheries research career	Spaven et al. (2009); hotline/outreach
LB24	Leatherback	Probable	Sighting	Alive	Sep in late 1970s or early 1980s	Southern BC	La Perouse Bank—bottom of Big Bank, 10 NM from CAN/US border, 15 NM off Cape Beale	48.567	−125.500	Exact			90	Observed while trolling for Coho salmon	Spaven et al. (2009); hotline/outreach
LB25	Leatherback	Certain	Sighting	Alive	Around 1980	Southern BC	Cape Cook—S of	49.987	−127.833	Best guess					Spaven et al. (2009, 2003); questionnaire
LB26	Leatherback	Certain	Sighting	Alive	Jul 1981	Southern BC	La Perouse Bank—off Ucluelet	48.756	−125.695	Best guess					hotline/outreach
LB27	Leatherback	Certain	Entanglement	Alive	30 Aug 1981	Northern BC	Skidegate Inlet—off Image Point	53.250	−131.958	Approximate	14.18			Released alive from commercial gillnet. Photos archived on file at Royal BC Museum (RBCM # 749)	Stinson (1984)
LB28	Leatherback	Certain	Sighting	Alive	2 Sep 1981	Northern BC	Skidegate Inlet	53.250	−131.950	Approximate	14.16	> 180		Observed with the released turtle from 30 Aug 1981, a few days post release	Stinson (1984)
LB29	Leatherback	Certain	Sighting	Alive	Sep 1981	Southern BC	Esperanza Point—15 NM SW of	49.710	−127.200	Best guess					Spaven et al. (2009, 2003); questionnaire
LB30	Leatherback	Certain	Sighting	Alive	16 Sep 1982	Southern BC	Esperanza Inlet	49.867	−126.733	Approximate	14.26			RBCM #826	McAlpine et al. (2004)
LB31	Leatherback	Certain	Sighting	Alive	17 Jul 1983	Northern BC	Graham Island—1.5 NM 332 deg NW of Selvesen Point	53.608	−133.022	Exact	13.33	~180			Stinson (1984)
LB32	Leatherback	Certain	Sighting	Alive	Between Jun and Aug of 1983	Southern BC	San Josef Bay, Brooks Peninsula—8 NM W of	50.633	−128.550	Approximate					Spaven et al. (2009); hotline/outreach
LB33	Leatherback	Probable	Sighting	Alive	Aug 1983	Southern BC	La Perouse Bank—offshore of	48.500	−125.167	Best guess		~240			Spaven et al. (2009, 2003); questionnaire
LB34	Leatherback	Certain	Sighting	Alive	Around 1983	Southern BC	Kains Island—10 NM off	50.333	−128.250	Best guess		< 150			Spaven et al. (2009, 2003); questionnaire
LB35	Leatherback	Probable	Sighting	Alive	Around Summer 1984	Northern BC	Hippa Island & Rennell Sound area	53.434	−132.767	Best guess					Spaven et al. (2009); hotline/outreach
LB36	Leatherback	Certain	Sighting	Alive	Sometime in 1984	Southern BC	Bull Harbour, Queen Charlotte Sound	50.900	−127.933	Approximate					Spaven et al. (2009); hotline/outreach
LB37	Leatherback	Certain	Mortality	Dead, skeletal remains	14 Feb 1985	Southern BC	Oak Bay, Victoria	48.427	−123.288	Approximate				Partial skeleton collected. No necropsy or further assessment. Retained by Royal BC Museum (#1612)	Spaven et al. (2009); Royal BC Museum
LB38	Leatherback	Certain	Sighting	Alive	1985 or 1986	Northern BC	Langara Island	54.217	−133.100	Best guess					Spaven et al. (2009); hotline/outreach
LB39	Leatherback	Certain	Sighting	Alive	Aug 1985	Southern BC	Vancouver Island—40 NM off	CBD	CBD	Uncertain					Spaven et al. (2009, 2003); questionnaire
LB40	Leatherback	Certain	Sighting	Alive	Around Aug 1985	Southern BC	Kains Island—5 NM off	50.383	−128.133	Approximate		< 150			Spaven et al. (2009, 2003); questionnaire
LB41	Leatherback	Certain	Sighting	Alive	Around 1985	Southern BC	Cape Cook—S of	49.917	−127.920	Best guess					Spaven et al. (2009, 2003); questionnaire
LB42	Leatherback	Certain	Sighting	Alive	Mid 1980s	Southern BC	Ucluelet Harbour—15 NM SW of	48.750	−125.750	Best guess					Spaven et al. (2009, 2003); questionnaire
LB43	Leatherback	Certain	Sighting	Alive	Mid 1980s	Southern BC	Ucluelet Harbour—SE of	48.667	−125.500	Best guess					Spaven et al. (2009, 2003); questionnaire
LB44	Leatherback	Probable	Entanglement	Alive	Jun or Jul around 1986	Northern BC	Skeena River—mouth of	54.117	−130.317	Approximate				Released alive from commercial gillnet	Spaven et al. (2009); hotline/outreach
LB45	Leatherback	Probable	Entanglement	Alive	10 Aug 1986	Southern BC	Nootka Sound—entrance to	49.533	−126.667	Approximate	14.26	~240		Flipper caught on commercial troll stabilizer line. Released alive with chain remaining on flipper	Spaven et al. (2009); hotline/outreach
LB46	Leatherback	Certain	Sighting	Alive	12 Aug 1986	Southern BC	Sea Otter Cove—30 NM S of	50.167	−128.333	Best guess		~250			Spaven et al. (2009, 2003); questionnaire
LB47	Leatherback	Probable	Sighting	Alive	Around 1986	Northern BC	Hippa Island & Rennell Sound area	53.440	−132.893	Best guess					Spaven et al. (2009); hotline/outreach
LB48	Leatherback	Certain	Sighting	Alive	Around 1988	Northern BC	Hippa Island & Rennell Sound area	53.400	−132.767	Best guess					Spaven et al. (2009); hotline/outreach
LB49	Leatherback	Probable	Entanglement	Alive	1 Sep 1989	Southern BC	Carmanah Point—2.4 NM offshore of lightstation	48.600	−124.820	Approximate	13.12			Released alive from commercial gillnet	Spaven et al. (2009); hotline/outreach
LB50	Leatherback	Certain	Sighting	Alive	Around 1989	Southern BC	Cape Beale—at “7 mile Bank”	48.667	−125.317	Approximate					Spaven et al. (2009, 2003); questionnaire
LB51	Leatherback	Probable	Mortality	Dead, unknown condition	Around 1989	Northern BC	Haida Gwaii—off one of the 3 islands on S tip	51.920	−131.000	Approximate				Not collected or examined	Spaven et al. (2009); hotline/outreach
LB52	Leatherback	Certain	Entanglement	Alive	Around 1989	Southern BC	Nitnat River mouth—off	48.633	−124.933	Approximate				Entangled and released alive from gillnet	Spaven et al. (2009); hotline/outreach
LB53	Leatherback	Probable	Sighting	Alive	Late 1980s	Northern BC	Haida Gwaii—W of	53.340	−132.851	Best guess		~240			2014 questionnaire
LB54	Leatherback	Probable	Sighting	Alive	Around 1990	Northern BC	Hippa Island & Rennell Sound area	53.483	−132.983	Best guess					Spaven et al. (2009); hotline/outreach
LB55	Leatherback	Certain	Sighting	Alive	Early 1990s	Southern BC	Cape Cook—6 NM off	50.117	−128.083	Best guess					Spaven et al. (2009, 2003); questionnaire
LB56	Leatherback	Certain	Sighting	Alive	Jul 1992	Southern BC	Triangle Island—S of	50.667	−129.083	Best guess		~140			Spaven et al. (2009, 2003); questionnaire
LB57	Leatherback	Certain	Sighting	Alive	Jul 1992	Southern BC	Estevan Point—10 NM S	49.200	−126.500	Best guess		~150			Spaven et al. (2009, 2003); questionnaire
LB58	Leatherback	Certain	Sighting	Alive	20 Aug 1992	Southern BC	Scott Islands—5 NM S of Triangle Island	50.750	−129.067	Approximate	15.07	~150			Spaven et al. (2009, 2003); questionnaire
LB59	Leatherback	Certain	Entanglement	Dead, unknown condition	Aug 1992	Northern BC	Cape St James—12 NM S of	51.704	−130.995	Best guess		~300		Found dead in crab gear. Reported in Spaven et al. (2009) with wrong date (was July 2001, but confirmed Aug 1992 is accurate)	Spaven et al. (2009, 2003); questionnaire
LB60	Leatherback	Certain	Sighting	Alive	Aug in early 1990s	Southern BC	Cape Beale—10 NM off	48.667	−125.417	Best guess					Spaven et al. (2009, 2003); questionnaire
LB61	Leatherback	Probable	Sighting	Alive	Oct 1992	Northern BC	Langara Island—18 NM NW off continental shelf	54.317	−133.600	Best guess		~200			Spaven et al. (2009); hotline/outreach
LB62	Leatherback	Certain	Sighting	Alive	Aug 1993	Southern BC	Brooks Peninsula—off Clerke Point	50.063	−127.817	Best guess				Observed feeding on Cyanea	McAlpine et al. (2004)
LB63	Leatherback	Certain	Sighting	Alive	9 Sep 1993	Southern BC	Esperanza Inlet—off	50.150	−129.817	Exact	14.07	~240			Spaven et al. (2009, 2003); questionnaire
LB64	Leatherback	Probable	Sighting	Alive	9 Aug 1994	Northern BC	Hecate Strait—approx 50 NM S of Sandspit's latitude	52.433	−130.433	Best guess		~200			Spaven et al. (2009); hotline/outreach
LB65	Leatherback	Certain	Sighting	Alive	26 May 1996	Southern BC	Victoria—off Mount Douglas Park	48.500	−123.300	Approximate	11				McAlpine et al. (2004)
LB66	Leatherback	Certain	Sighting	Alive	Jun 1996	Southern BC	Cape Scott—2 NM SW of	50.167	−128.467	Approximate	11.92	~150			Spaven et al. (2009, 2003); questionnaire
LB67	Leatherback	Certain	Sighting	Alive	10 Jul 1996	Central BC	Queen Charlotte Sound	51.300	−129.033	Best guess					Spaven et al. (2009); WBT Wildlife Data Centre
LB68	Leatherback	Certain	Sighting	Alive	Aug 1996	Southern BC	Brooks Peninsula—E side near the Shelter Shed	50.066	−127.733	Approximate	13.55			Turtle eating an orange jellyfish	Spaven et al. (2009, 2003); questionnaire
LB69	Leatherback	Certain	Sighting	Alive	Aug 1996	Southern BC	Wilf Rock, Clayoquot Sound—0.5 NM off	49.135	−125.978	Approximate					2014 questionnaire
LB70	Leatherback	Certain	Sighting	Alive	Sep 1996	Southern BC	Cape Cook—50 NM off on tuna grounds	50.083	−129.250	Best guess		~180	~100		Spaven et al. (2009, 2003); questionnaire
LB71	Leatherback	Certain	Mortality	Dead, fresh	May 1997	Southern BC	Kyuquot	50.083	−127.217	Approximate				Necropsy by school group. Samples collected and some bones retained. Unable to locate specimens. Unknown post mortem results. Photos	McAlpine et al. (2004)
LB72	Leatherback	Probable	Sighting	Alive	Mid Jul 1997	Southern BC	Cape Beale—10‐12 NM SW offshore	48.617	−125.350	Best guess		> 240	~145		Spaven et al. (2009); hotline/outreach
LB73	Leatherback	Certain	Sighting	Alive	Sep 1997	Northern BC	Langara Island—SE side	54.200	−132.967	Approximate	13.52	~240			McAlpine et al. (2004)
LB74	Leatherback	Certain	Mortality	Dead, moderate decomposition	1 Feb 1998 (or 1997)	Northern BC	Dixon Entrance—inside the “Gully”	54.250	−131.025	Best guess		> 90		Moderately decomposed carcass caught by dragger. Conditon of carcass indicates COD was not the dragger	Spaven et al. (2009); hotline/outreach
LB75	Leatherback	Certain	Mortality	Dead, advanced decomposition	Spring 1998	Southern BC	Tonquin Beach, Esowista Peninsula	49.135	−125.911	Approximate				Not collected or examined	McAlpine et al. (2004)
LB76	Leatherback	Probable	Sighting	Alive	20 Apr 1999	Southern BC	Clayoquot Sound—W of Cleland Island	49.167	−126.083	Best guess					Spaven et al. (2009); WBT Wildlife Data Centre
LB77	Leatherback	Certain	Sighting	Alive	10 Jun 1999	Southern BC	Vancouver Island—55 NM SW of Carmanah Point at the CAN/US border	48.167	−125.950	Best guess					Spaven et al. (2009); hotline/outreach
LB78	Leatherback	Certain	Vessel strike	Alive	24 Jul 1999	Southern BC	Kirby Point, Barkley Sound	48.850	−125.208	Approximate	13.33	~200		Struck by boat trolling at slow speed. Turtle was not likely injured	Spaven et al. (2009); hotline/outreach
LB79	Leatherback	Certain	Sighting	Alive	1990s	Southern BC	Carmanah Point—towards Cape Beale	48.668	−124.992	Best guess				More than 1 turtle observed in the 1990s	2014 questionnaire
LB80	Leatherback	Probable	Sighting	Alive	27 Jun 2000	Southern BC	Clayquot Sound—3 NM off Vargas Island	49.133	−126.083	Approximate	12.39	~100			Spaven et al. (2009); hotline/outreach
LB81	Leatherback	Certain	Sighting	Alive	Aug 2000	Northern BC	Haida Gwaii—off W Cape St James	51.883	−130.867	Best guess		~240			Spaven et al. (2009, 2003); questionnaire
LB82	Leatherback	Certain	Sighting	Alive	Aug 2000	Southern BC	Top Knot Point—60 NM S of	49.533	−128.217	Best guess		~240			Spaven et al. (2009, 2003); questionnaire
LB83	Leatherback	Certain	Sighting	Alive	6 Sep 2000	Southern BC	Estevan Point—55 NM WSW of	48.720	−127.433	Exact	14.87				McAlpine et al. (2004)
LB84	Leatherback	Certain	Sighting	Alive	Apr 2001	Southern BC	Friendly Cove, Nootka Sound	49.583	−126.600	Approximate	8.79				Spaven et al. (2009); hotline/outreach
LB85	Leatherback	Certain	Mortality	Dead, unknown condition	Jul 2001	Southern BC	Tonquin Beach—off	49.116	−125.895	Approximate				Not collected or examined	Spaven et al. (2009, 2003); questionnaire
LB86	Leatherback	Certain	Sighting	Alive	4 Aug 2001	Northern BC	Langara Island—off	54.300	−133.167	Best guess				Photos	McAlpine et al. (2004)
LB87	Leatherback	Certain	Mortality	Dead, moderate decomposition	12 Aug 2001	Northern BC	Darwin Sound—near Shuttle Island	52.650	−131.667	Approximate		~200		Not collected or examined. Photos	Spaven et al. (2009, 2003); questionnaire
LB88	Leatherback	Probable	Sighting	Alive	8 Sep 2001	Southern BC	Quatsino Sound—Rupert Inlet, in small bay between camp and log sort	50.578	−127.508	Approximate	12.92		~60		Spaven et al. (2009); hotline/outreach
LB89	Leatherback	Certain	Sighting	Alive	Sep 2001	Southern BC	Dellwood Knoll—75 NM W of Cape Scott	50.933	−130.500	Best guess					2014 questionnaire
LB90	Leatherback	Certain	Sighting	Alive	Jun 2002	Southern BC	Nootka Island—35 NM off	49.250	−127.083	Best guess		~200			Spaven et al. (2009, 2003); questionnaire
LB91	Leatherback	Certain	Sighting	Alive	29 Jul 2003	Northern BC	Laskeek Bay—10 NM S of Reef Island	52.700	−131.383	Best guess					Spaven et al. (2009); hotline/outreach
LB92	Leatherback	Certain	Sighting	Alive	6 Aug 2003	Northern BC	Haida Gwaii—SE of	52.337	−130.954	Exact	15.18				Spaven et al. (2009); hotline/outreach
LB93	Leatherback	Probable	Sighting	Alive	8 Aug 2003	Southern BC	Denman Island—off Sandy Island	49.617	−124.833	Approximate	15.63				Spaven et al. (2009); hotline/outreach
LB94	Leatherback	Certain	Sighting	Alive	11 Aug 2003	Northern BC	Juan Perez Sound—3 NM off of NE corner of Murchison Island	52.617	−131.333	Approximate	15.2				Spaven et al. (2009); hotline/outreach
LB95	Leatherback	Certain	Sighting	Alive	Aug 2003	Southern BC	Cape Beale—45 NM SW of	48.167	−125.917	Best guess		~150			Spaven et al. (2009, 2003); questionnaire
LB96	Leatherback	Certain	Sighting	Alive	Aug 2003	Southern BC	Vancouver Island—W of	CBD	CBD	Uncertain					Spaven et al. (2009, 2003); questionnaire
LB97	Leatherback	Probable	Sighting	Alive	30 May 2004	Southern BC	Sooke Harbour—towards Otter Point	48.363	−123.792	Approximate	12.99	~200			Spaven et al. (2009); hotline/outreach
LB98	Leatherback	Probable	Sighting	Alive	21 Jun 2004	Northern BC	Gospel Island, Rennel Sound—1 NM W of	53.383	−132.633	Approximate	11.84	~240	~170		Spaven et al. (2009); hotline/outreach
LB99	Leatherback	Certain	Sighting	Alive	6 Jul 2004	Southern BC	Pine Island—1 NM W of	50.967	−127.750	Approximate	14.8	~300			Spaven et al. (2009); hotline/outreach
LB100	Leatherback	Certain	Sighting	Alive	9 Jul 2004	Northern BC	Lost Island—1 NM E of	52.800	−131.400	Approximate	14.76	~150			Spaven et al. (2009); hotline/outreach
LB101	Leatherback	Certain	Sighting	Alive	20 Jul 2004	Northern BC	Port Louis—towards Hippa Island	53.617	−133.050	Best guess		~210	~120	Photos	Spaven et al. (2009); hotline/outreach
LB102	Leatherback	Certain	Sighting	Alive	28 Jul 2004	Southern BC	Pachena Point—70 NM W of	48.713	−126.908	Exact	15.52	> 180	~140		Spaven et al. (2009); hotline/outreach
LB103	Leatherback	Certain	Sighting	Alive	9 Aug 2004	Northern BC	Langara Island—1 NM W of	54.233	−133.117	Approximate	15.47			Photos	Spaven et al. (2009); hotline/outreach
LB104	Leatherback	Certain	Sighting	Alive	16 Aug 2004	Southern BC	Esperanza Inlet—50 NM W of	49.733	−128.333	Best guess					Spaven et al. (2009); hotline/outreach
LB105	Leatherback	Certain	Sighting	Alive	7 Sep 2004	Southern BC	French Beach—near	48.367	−123.950	Approximate	13.94	> 170			Spaven et al. (2009); hotline/outreach
LB106	Leatherback	Probable	Mortality	Dead, unknown condition	25 Sep 2004	Southern BC	Amphitrite Point—10 NM S of	48.752	−125.520	Best guess		~150	~90	Not collected or examined	Spaven et al. (2009); hotline/outreach
LB107	Leatherback	Certain	Sighting	Alive	26 Aug 2005	Northern BC	Houston Stewart Channel—W of	51.962	−131.303	Best guess		~130		Photos	Spaven et al. (2009); vessel survey
LB108	Leatherback	Certain	Sighting	Alive	11 Sep 2005	Northern BC	Langara Island—E of	54.250	−132.917	Best guess					Spaven et al. (2009); hotline/outreach
LB109	Leatherback	Certain	Sighting	Alive	15 Sep 2005	Southern BC	Dellwood Seamount—100 NM W of Triangle Island	50.320	−130.942	Best guess					Spaven et al. (2009); hotline/outreach
LB110	Leatherback	Probable	Sighting	Alive	16 Sep 2005	Southern BC	Dellwood Knolls—W of	50.583	−130.667	Best guess				More than 1 turtle observed while tuna fishing	Spaven et al. (2009); hotline/outreach
LB111	Leatherback	Certain	Sighting	Alive	16 Sep 2005	Southern BC	Dellwood Seamount—100 NM W of Triangle Island	50.312	−131.235	Best guess					Spaven et al. (2009); hotline/outreach
LB112	Leatherback	Certain	Sighting	Alive	17 Sep 2005	Southern BC	Dellwood Seamount—100 NM W of Triangle Island	50.331	−130.772	Best guess					Spaven et al. (2009); hotline/outreach
LB113	Leatherback	Certain	Sighting	Alive	11 Aug 2007	Central BC	Queen Charlotte Sound	51.350	−131.167	Exact	15.47				Spaven et al. (2009); vessel survey
LB114	Leatherback	Certain	Sighting	Alive	Oct 2007	Southern BC	Galleon Beach, Hornby Island	49.550	−124.667	Approximate	11.27	~150			Spaven et al. (2009); hotline/outreach
LB115	Leatherback	Certain	Sighting	Alive	15 Jul 2008	Northern BC	Frederick Island—near	53.930	−133.217	Best guess					2014 questionnaire
LB116	Leatherback	Certain	Sighting	Alive	7 Aug 2008	Southern BC	Cape Beale—80 NM SW of	48.003	−127.050	Exact	14.9				Spaven et al. (2009); hotline/outreach
LB117	Leatherback	Probable	Sighting	Alive	27 Aug 2008	Southern BC	La Perouse Bank—11 NM off Lennard Island Lightstation	49.017	−126.167	Best guess	13.42	~150			Spaven et al. (2009); hotline/outreach
LB118	Leatherback	Certain	Sighting	Alive	4 Sep 2008	Southern BC	La Perouse Bank—20 NM W of Barkley Sound	48.704	−125.824	Exact					Spaven et al. (2009); hotline/outreach
LB119	Leatherback	Probable	Sighting	Alive	25 Sep 2008	Southern BC	Wickaninnish Bay—off Portland Point	49.006	−125.824	Approximate		150			Spaven et al. (2009); hotline/outreach
LB120	Leatherback	Certain	Sighting	Alive	Sep 2008	Southern BC	Nootka Sound—85 NM off	49.500	−129.000	Best guess					2014 questionnaire
LB121	Leatherback	Probable	Sighting	Alive	Sometime in 2008	Southern BC	Vancouver Island— S	CBD	CBD	Uncertain					2014 questionnaire
LB122	Leatherback	Certain	Sighting	Alive	11 Sep 2009	Southern BC	Tofino—70 NM SW of	48.356	−129.317	Exact	15.64				Spaven et al. (2009); hotline/outreach
LB123	Leatherback	Certain	Sighting	Alive	6 Jul 2010	Southern BC	Swiftsure Bank— 10‐14 NM from shore, just inside J buoy	48.495	−124.918	Approximate	13.15	~200			Hotline/outreach
LB124	Leatherback	Certain	Sighting	Alive	10 Aug 2010	Northern BC	Hippa Island—near	53.527	−133.002	Best guess					2014 questionnaire
LB125	Leatherback	Certain	Sighting	Alive	9 Sep 2010	Southern BC	Quatsino Sound—near Kains Island	50.308	−128.222	Approximate	13.99	~210	~120		Hotline/outreach
LB126	Leatherback	Certain	Sighting	Alive	17 May 2011	Southern BC	Bamfield Inlet— in front of the Bamfield Marine Science Center	48.836	−125.136	Exact	10.45				Hotline/outreach
LB127	Leatherback	Certain	Sighting	Alive	1 Aug 2011	Southern BC	Clayoquot Canyon—25 NM offshore at the mouth of	48.920	−126.555	Best guess					Hotline/outreach
LB128	Leatherback	Certain	Sighting	Alive	20 Aug 2011	Southern BC	LaPerouse Bank	48.815	−125.847	Best guess		> 100			Hotline/outreach
LB129	Leatherback	Probable	Sighting	Alive	21 Jun 2012	Southern BC	Georgia Strait— N of Salmon Point	49.899	−125.127	Best guess					Hotline/outreach
LB130	Leatherback	Certain	Sighting	Alive	18 Aug 2012	Southern BC	Cape Flaherty—70 NM W along CAN/US border	48.399	−126.492	Best guess		> 120			Hotline/outreach
LB131	Leatherback	Certain	Mortality	Dead, advanced decomposition	21 Aug 2012	Northern BC	Queen Charlotte Sound—2 NM S of Cape St James	51.895	−131.003	Approximate				Not collected or examined. Photos	Hotline/outreach
LB132	Leatherback	Certain	Sighting	Alive	5 Aug 2013	Southern BC	Quatsino Sound—1 NM off Lippy Point	50.462	−128.130	Approximate	15.44	~230			Hotline/outreach
LB133	Leatherback	Certain	Sighting	Alive	8 Aug 2013	Southern BC	Quatsino Sound—2‐3 NM off Lippy Point	50.449	−128.148	Approximate	15.44				Hotline/outreach
LB134	Leatherback	Certain	Sighting	Alive	11 Sep 2013	Southern BC	Barkley Sound—80 NM W of	48.825	−127.437	Exact	16.68		120		2014 questionnaire
LB135	Leatherback	Probable	Sighting	Alive	14 Sep 2013	Southern BC	Kains Island, Quatsino Sound—32 NM off	50.078	−128.639	Best guess					Hotline/outreach
LB136	Leatherback	Certain	Sighting	Alive	15 Sep 2013	Southern BC	Cape Alava, Washington—100 NM W of (in BC waters)	48.170	−127.760	Exact	16.68		< 100	Observed consuming a jellyfish	2014 questionnaire
LB137	Leatherback	Certain	Sighting	Alive	20 Aug 2014	Southern BC	Tofino—near La Croix Group Islands	49.137	−125.977	Approximate		~210		Photos	Hotline/outreach
LB138	Leatherback	Certain	Sighting	Alive	Between Aug and Sep of 2014	Southern BC	Cape Cook—towards Cape Scott	50.566	−128.478	Best guess					2014 questionnaire
LB139	Leatherback	Certain	Sighting	Alive	25 Jul 2016	Southern BC	Brooks Peninsula—72.5 NM SW of	49.567	−129.604	Exact	15.4				Vessel survey
LB140	Leatherback	Certain	Sighting	Alive	1‐Jul‐2017	Southern BC	Brooks Peninsula—1 NM NW of	50.138	−127.933	Best guess					Hotline/outreach
LB141	Leatherback	Certain	Sighting	Alive	1‐Aug‐2017	Southern BC	Kyuquot Sound	49.986	−127.330	Approximate	15.5				Hotline/outreach
LB142	Leatherback	Certain	Sighting	Alive	6 Aug 2018	Southern BC	Lowden Canyon	48.682	−126.302	Exact	15.57			Photos	Hotline/outreach
LB143	Leatherback	Certain	Sighting	Alive	Sep 4 2019	Northern BC	Finlayson Channel at Tolmie Channe— at the tip of Sarah Island	52.643	−128.53	Exact	14.81				Hotline/outreach
LB144	Leatherback	Certain	Sighting	Alive	11 Sep 2020	Southern BC	South West of Brooks Peninsula	49.626	−128.099	Exact	16.19			Photos	Hotline/outreach
LB145	Leatherback	Probable	Sighting	Alive	19 Aug 2022	Southern BC	Swiftsure Bank	48.442	−126.158	Exact	16.1				Hotline/outreach
LB146	Leatherback	Probable	Entanglement	Alive	Unknown date	Northern BC	Hippa Island—near Nestle Island	53.533	−132.933	Approximate				Released alive from seine net	Spaven et al. (2009); hotline/outreach
LB147	Leatherback	Probable	Sighting	Alive	Unknown date	Northern BC	Houston Stewart Channel	52.150	−131.000	Approximate					Spaven et al. (2009); hotline/outreach
LB148	Leatherback	Probable	Entanglement	Alive	Unknown date	Northern BC	Langara Island—near	54.300	−133.083	Best guess				Released alive from seine net	Spaven et al. (2009); hotline/outreach
**Green sea turtle**															
G1	Green	Certain	Live stranding	Alive, died	6 Dec 1954	Southern BC	Spring Cove, Barkely Sound—W entrance to	48.926	−125.530	Approximate	10.39	47		Held in temporary containment at head of Alberni canal; died later that week. No necropsy or further assessment	Carl (1955)
G2	Green	Certain	Sighting	Alive	Apr or May 1958	Southern BC	Nootka Sound	49.500	−126.650	Best guess					Radovich (1960)
G3	Green	Probable	Sighting	Alive	Early 1980s	Southern BC	Cape Flattery—20 NM off	48.400	−125.180	Best guess					2003 questionnaire
G4	Green	Certain	Sighting	Alive	5 Sep 1981	Southern BC	Carmanah Point—offshore	49.125	−127.130	Best guess				Two turtles seen 50 NM from each other on same day	Stinson (1984)
G5	Green	Certain	Sighting	Alive	Sep 1981	Northern BC	Chatham Sound—E of Dundas Island	54.500	−130.670	Approximate	13.65			Multiple reported sightings of live green sea turtles from this date at this location	Hodge and Wing (2000)
G6	Green	Probable	Sighting	Alive	Sep 1981	Southern BC	Esteven Point—30 NM W of	49.376	−127.330	Best guess					Hotline/outreach
G7	Green	Certain	Sighting	Alive	17 Oct 1981	Southern BC	Pachena Point—0.5 NM offshore of lightstation	48.705	−125.117	Approximate	14.19	~100		2 live turtles seen near net set	Stinson (1984)
G8	Green	Certain	Mortality	Dead, advanced decomposition	25 Apr 1994	Southern BC	Schooner Cove, Pacific Rim National Park Reserve	49.067	−125.800	Exact		~60		External exam conducted. Photos	McAlpine et al. (2002)
G9	Green	Certain	Mortality	Dead, skeletal remains	22 Nov 1996	Northern BC	Hartley Bay	53.420	−129.250	Approximate				Not collected or examined. Photos	McAlpine et al. (2004)
G10	Green	Certain	Mortality	Dead, unknown condition	28 Feb 1997	Southern BC	Ucluth Penninsula—near “Garbage Dump” beaches N of Ucluelet	48.950	−125.583	Approximate		~100		Not collected or examined	McAlpine et al. (2002)
G11	Green	Probable	Sighting	Alive	Nov 1998	Northern BC	Cloak Bay, Langara Island	54.212	−133.030	Exact	9.62	~65	~65		Hotline/outreach
G12	Green	Certain	Mortality	Dead, unknown condition	Early Nov 1998	Northern BC	Tlell River—2.5 NM S of	53.583	−131.900	Approximate				Another report from same month at similar location but reported as 2 separate sightings in McAlpine et al.	McAlpine et al. (2004)
G13	Green	Probable	Mortality	Dead, unknown condition	Early Nov 1998	Northern BC	Tlell River—near mouth	53.600	−131.933	Approximate				Another report from same month at similar location but reported as 2 separate sightings in McAlpine et al.	McAlpine et al. (2004)
G14	Green	Certain	Mortality	Dead, fresh	9 Nov 1999	Northern BC	Rose Point—2.5 NM S of	54.200	−131.633	Approximate		~71		May have been alive at time of initial stranding. Could be same as 3 other reports of carcass at similar location the same month but reported as separate sightings in McAlpine et al. No necropsy or further assessment	McAlpine et al. (2002)
G15	Green	Certain	Mortality	Dead, fresh	16 Nov 1999	Northern BC	Rose Point—towards Fife Point	54.100	−131.667	Approximate		~81		Could be same as 3 other reports of carcass at similar location the same month but reported as separate sightings in McAlpine et al. No necropsy or further assessment	McAlpine et al. (2002)
G16	Green	Certain	Mortality	Dead, unknown condition	Late Nov 1999	Northern BC	Rose Point—17 NM S of	54.017	−131.717	Best guess		~81		Could be same as 3 other reports of carcass at similar location the same month but reported as separate sightings in McAlpine et al. No necropsy or further assessment	McAlpine et al. (2002)
G17	Green	Certain	Mortality	Dead, unknown condition	28 Jan 2000	Southern BC	Esquimalt Lagoon, Metchosin	48.420	−123.460	Approximate		87		Remains collected. No necropsy or further assessment	McAlpine et al. (2002)
G18	Green	Certain	Mortality	Dead, unknown condition	Early Jan 2000	Northern BC	Rose Point—17 NM S of	54.000	−131.717	Best guess				Could be same as 3 other reports of carcass at similar location the same month but reported as separate sightings in McAlpine et al. No necropsy or further assessment	McAlpine et al. (2002)
G19	Green	Certain	Mortality	Dead, fresh	6 Nov 2001	Northern BC	Tlell River—2 NM N of	53.370	−131.800	Approximate		78.7		Not collected or examined	McAlpine et al. (2004)
G20	Green	Certain	Mortality	Dead, moderate decomposition	20 Dec 2001	Southern BC	Pacific Rim National Park Reserve—off Green Point	49.050	−125.717	Exact		68.7		Collected for necropsy. COD: trauma, secondary sepsis, cold stress. Photos	McAlpine et al. (2004)
G21	Green	Certain	Mortality	Dead, advanced decomposition	21 Jan 2002	Southern BC	Estevan Point—towards Matlahaw Point	49.383	−126.483	Exact		69.9		Collected for necropsy. COD: could not be determined due to post mortem change	McAlpine et al. (2004)
G22	Green	Certain	Live stranding	Alive	31 Aug 2005	Central BC	Schooner Pass— near GitxaalaVillage	53.750	−130.400	Approximate	15.61	84	61	Collected for rehabilitation. Symptoms of cold shock. Animal permenantly housed in captivity, originally at Vancouver Aquarium then transferred to Ripley's Aquarium in Toronto in 2023. Named “Schoona.” Photos	Hotline/outreach
G23	Green	Certain	Mortality	Dead, fresh	28 Jun 2007	Northern BC	Goose Island	51.909	−128.480	Exact		57		Not collected or examined. Photos	2014 questionnaire
G24	Green	Probable	Sighting	Alive	7 Aug 2009	Southern BC	Kyuquot Sound—1 NM N of Thornton Islands	49.988	−127.348	Approximate	14.44				Hotline/outreach
G25	Green	Certain	Mortality	Dead, skeletal remains	4 May 2010	Southern BC	Flores Island—near	49.385	−126.171	Approximate				Carapace and some tissues collected. No necropsy or further assessment. Genetic confirmation to Eastern Pacific population. Photos	Hotline/outreach
G26	Green	Certain	Live stranding	Alive, died	30 Nov 2011	Southern BC	Combers Beach, Pacific Rim National Park Reserve	49.041	−125.707	Approximate				Collected for rehabilitation. Died‐necropsy completed. COD: probable cold shock. Superficial trauma. Genetic confirmation to Eastern Pacific population. Photos	Hotline/outreach
G27	Green	Certain	Live stranding	Alive, died	7 Dec 2011	Southern BC	Green Point, Pacific Rim National Park Reserve	49.050	−125.720	Approximate		102	73	Collected for rehabilitation. Died‐necropsy completed. COD: Cold shock. Genetic confirmation to Eastern Pacific population. Photos	Hotline/outreach
G28	Green	Certain	Mortality	Dead, moderate decomposition	5 Feb 2012	Southern BC	Wickaninnish Beach, Pacific Rim National Park Reserve	49.015	−125.675	Approximate				Necropsy completed. COD: trauma with secondary septicemia. Genetic confirmation to Eastern Pacific population. Photos	Hotline/outreach
G29	Green	Certain	Mortality	Dead, advanced decomposition	9 Feb 2013	Southern BC	Juan de Fuca Strait—W end of Cribs Beach near Carmanah lightstation	48.630	−124.776	Approximate		57	45	Tissue collected. No necropsy or further assessment. Photos	Hotline/outreach
G30	Green	Certain	Mortality	Dead, moderate decomposition	29 Nov 2014	Northern BC	Dogfish Bank	53.947	−131.719	Best guess		32	26	Necropsy completed. COD: Could not be determined due to post mortem change	Hotline/outreach
G31	Green	Certain	Live stranding	Alive	23 Jan 2016	Southern BC	Combers Beach, Pacific Rim National Park Reserve	49.036	−125.701	Approximate		67	52	Collected for rehabilitation. Symptoms of cold shock. Released off San Diego in 2016. Named “Comber.” Photos	Hotline/outreach
G32	Green	Probable	Mortality	Dead, advanced decomposition	8 Nov 2024	Northern BC	Tlell, on nearby beach			Approximate				Not collected or examined. Photos.	Hotline/outreach
**Loggerhead sea turtle**															
LH1	Loggerhead	Certain	Sighting	Alive	22 Feb 2015	Southern BC	Barkely Sound—50 NM W of	48.670	−126.880	Exact	10.51			Photos	Halpin et al. (2018)
LH2	Loggerhead	Certain	Live stranding	alive	2 Feb 2024	Southern BC	Pedder Bay, Mechosin	48.333	−123.533	Approximate	9.22	63	52	Collected for rehabilitation. Named “Moira.” Cold shock. To be released off San Diego in 2025. Photos	Hotline/outreach
LH3	Loggerhead	Certain	Mortality	Dead, moderate decomposition	5 Apr 2024	Southern BC	Cape Scott	50.783	128.3633	Exact				Not collected or examined. Photos	Hotline/outreach
**Olive ridley sea turtle**															
OR1	Olive Ridley	Certain	Live stranding	Alive, died	22 Nov 2011	Southern BC	Wickaninnish Beach, Pacific Rim National Park Reserve	49.021	−125.680	Approximate		70		Collected for rehabilitation. Died‐necropsy completed. COD: acute trauma, muscular hemorrhage, probable cold shock. Plastic ingestion. Genetic confirmation to Eastern Pacific population. Photos	Hotline/outreach
OR2	Olive Ridley	Certain	Mortality	Dead, moderate decomposition	25 Oct 2013	Northern BC	Hecate Strait	54.087	−131.304	Approximate		~90		Necropsy completed. COD: possible cold shock. Photos	Hotline/outreach
OR3	Olive Ridley	Certain	Mortality	Dead, moderate decomposition	1 Feb 2015	Southern BC	Pacific Rim National Park Reserve	49.000	−125.750	Approximate				Necropsy completed. COD: possible cold shock. Photos	Hotline/outreach
OR4	Olive Ridley	Certain	Live stranding	Alive	Sep 30 2019	Southern BC	Port Alberni, 3500 Harbour Rd	49.238	−124.810	Approximate	14.92	64	59	Collected for rehabilitation. Symptoms of cold shock. Released off San Diego in 2020. Named “Berni.” Photos	Hotline/outreach
OR5	Olive Ridley	Certain	Sighting	Alive	27 Oct 2023	Southern BC	Tofino—3.5 nm offshore of Cleland Island	49.127	−126.151	Exact	13.69	~55		Photos	Hotline/outreach
**Unidentified hard‐shelled sea turtle**															
UHS1	Unidentified hard shelled sea turtle		Sighting	Alive	Mid 1960s	Southern BC	Cape Cook—towards Cape Scott	50.450	−128.350	Best guess				Large and green in colour	Spaven et al. (2009, 2003); questionnaire
UHS2	Unidentified hard shelled sea turtle		Entanglement	Alive	1976 or 1977	Southern BC	Cape Calvert, Fitz Hugh Sound	51.409	−127.857	Approximate				Released alive from gillnet. Large and green in colour	Spaven et al. (2009); hotline/outreach
UHS3	Unidentified hard shelled sea turtle		Sighting	Alive	Mid 1980s	Southern BC	Cape Beale—midway to Cape Flattery	48.520	−124.890	Best guess					2003 questionnaire
UHS4	Unidentified hard shelled sea turtle		Sighting	Alive	Jul or Aug of 1988 or 1989	Southern BC	Cape Flattery—33 NM W of	48.333	−125.500	Best guess					Spaven et al. (2009); hotline/outreach
UHS5	Unidentified hard shelled sea turtle		Sighting	Alive	Aug 1990	Southern BC	Brooks Bay—near Cape Cook	50.241	−127.915	Approximate	15.8				2014 questionnaire
UHS6	Unidentified hard shelled sea turtle		Sighting	Alive	Around 1994	Southern BC	Hope Island and Nahwitti Bar—N of	50.967	−128.133	Approximate				Green‐brown colour	Hotline/outreach
UHS7	Unidentified hard shelled sea turtle		Mortality	Dead, unknown condition	Mid 1990s	Northern BC	Howe Bay Beach	52.016	−131.035	Approximate				Not collected or examined	2014 questionnaire
UHS8	Unidentified hard shelled sea turtle		Sighting	Alive	Aug 1997	Southern BC	Quatsino Sound—off Kain's Island	50.430	−128.040	Approximate	15.79			Appeared to be feeding on small schooling fish like herring or sandlance	Spaven et al. (2009, 2003); questionnaire
UHS9	Unidentified hard shelled sea turtle		Mortality	Dead, skeletal remains	6 Jun 1998 (or 1997)	Northern BC	Caamano Sound—near Rennison Island	52.830	−129.369	Approximate		< 100		Bones collected. No necropsy or further assessment	Spaven et al. (2009); hotline/outreach
UHS10	Unidentified hard shelled sea turtle		Sighting	Alive	22 Sep 1998	Southern BC	Nitinat River mouth bar—W of	48.666	−124.897	Approximate	13.68	~75		Brown colour	2014 questionnaire
UHS11	Unidentified hard shelled sea turtle		Sighting	Alive	Nov 1998	Northern BC	Laredo Channel—off Aristazabal Island	52.383	−128.850	Approximate	10.7			Found turtle comatose on sea floor. Rewarmed in sun and released alive. Photos	Hotline/outreach
UHS12	Unidentified hard shelled sea turtle		Mortality	Dead, skeletal remains	15 Nov 2001	Southern BC	Juan de Fuca Strait—off Sombrio Beach	48.474	−124.258	Approximate		~100		Not collected or examined	Hotline/outreach
UHS13	Unidentified hard shelled sea turtle		Sighting	Alive	28 Aug 2003	Southern BC	West Vancouver—off Ambleside Park	49.326	−123.162	Exact	15.69	> 60			Hotline/outreach
UHS14	Unidentified hard shelled sea turtle		Mortality	Dead, skeletal remains	20 Jun 2005	Northern BC	Prince Rupert—near	53.850	−130.667	Exact				Plastron collected. No necropsy or further assessment. Retained by Royal BC Museum. DNA extraction unable to amplify—no ID possible	Royal BC Museum
UHS15	Unidentified hard shelled sea turtle		Sighting	Alive	5 May 2008	Southern BC	Jervis Inlet—midway between Hardy Island and Saltery Bay	49.770	−124.173	Approximate	9.92	> 100		Brown shell, slim head extended 50cm in front of shell	Hotline/outreach
UHS16	Unidentified hard shelled sea turtle		Sighting	Alive	29 Jul 2012	Southern BC	Pendrell Sound—entrance of	50.207	−124.753	Approximate				Greenish brown, tear drop shape, reddish brown streaking on the underside. Slender front flippers	Hotline/outreach
UHS17	Unidentified hard shelled sea turtle		Sighting	Alive	9 Nov 2015	Southern BC	Florencia Bay—S entrance near Wya Point	48.974	−125.626	Exact	12.54	~125		Smooth chocolate brown carapace	Hotline/outreach
UHS18	Unidentified hard shelled sea turtle		Sighting	Alive	30 Aug 2017	Southern BC	Shadwell Pass, Queen Charlotte Strait	50.946	−127.81	Exact	14.98	~90	~60	Seen gliding up underwater while observering a fried egg jelly. Turtle was slightly oblong in shape and yellowish brown colour	Vessel survey
UHS19	Unidentified hard shelled sea turtle		Sighting	Alive	4 Aug 2022	Northern BC	Prince Rupert	54.321	−130.317	Exact	14.99				Hotline/outreach
UHS20	Unidentified hard shelled sea turtle		Sighting	Alive	7 Jul 2024	Southern BC	Green Point, Pacific Rim National Park Reserve	49.058	−125.759	Exact				Oval shaped and the size of a standard cooler	Hotline/outreach
**Unidentified sea turtle**															
U1	Unidentified sea turtle		Entanglement	Alive	Jun or Jul between 1958 and 1963	Central BC	Rivers Inlet—near Major Brown Rock Reef and Bull‐Sharbow Islands	51.422	−127.713	Approximate				Released alive from commercial gillnet	2014 questionnaire
U2	Unidentified sea turtle		Sighting	Alive	Around 1974	Southern BC	Brooks Peninsula—6 NM off	50.012	−128.000	Best guess					2003 questionnaire
U3	Unidentified sea turtle		Sighting	Alive	Around Sep 1979	Northern BC	Skidegate Channel	53.217	−131.967	Approximate					Spaven et al. (2009, 2003); questionnaire
U4	Unidentified sea turtle		sighting	Alive	Aug or Sep around 1984	Southern BC	Vancouver Island—off NW	CBD	CBD	Uncertain					Spaven et al. (2009, 2003); questionnaire
U5	Unidentified sea turtle		Mortality	Dead, unknown condition	Mid 1980s	Southern BC	Ahousat, Clayoquot Sound—near	49.267	−126.033	Approximate		~180		Not collected or examined	Spaven et al. (2009); hotline/outreach
U6	Unidentified sea turtle		Sighting	Alive	Mid 1980s	Northern BC	Haida Gwaii—W of	53.000	−133.000	Best guess					Spaven et al. (2009, 2003); questionnaire
U7	Unidentified sea turtle		Sighting	Alive	1 Jul 1987	Northern BC	Haida Gwaii—W of	53.030	−133.050	Best guess					Spaven et al. (2009); hotline/outreach
U8	Unidentified sea turtle		Sighting	Alive	Around 1989	Southern BC	Juan de Fuca Strait	48.417	−124.333	Best guess			~120		Spaven et al. (2009, 2003); questionnaire
U9	Unidentified sea turtle		Sighting	Alive	Early 1990s	Southern BC	Vancouver Island—W of mid island	CBD	CBD	Uncertain		~250		Very large	Spaven et al. (2009); hotline/outreach
U10	Unidentified sea turtle		Sighting	Alive	Mid 1990s	Southern BC	Brooks Peninsula—15 NM off	49.940	−128.180	Best guess					2003 questionnaire
U11	Unidentified sea turtle		Sighting	Alive	Aug 1991	Southern BC	Vancouver Island—off NW	CBD	CBD	Uncertain				Very large	Spaven et al. (2009, 2003); questionnaire
U12	Unidentified sea turtle		Sighting	Alive	Sometime in 1992	Central BC	Goose Bank—SW corner	51.417	−129.333	Best guess					Spaven et al. (2009, 2003); questionnaire
U13	Unidentified sea turtle		Sighting	Alive	Jul 1994	Southern BC	Brooks Peninsula	50.183	−127.967	Best guess					Spaven et al. (2009); hotline/outreach
U14	Unidentified sea turtle		Sighting	Alive	Sep 1994	Northern BC	Cumshewa Inlet—mouth of	53.006	−131.610	Approximate	13.27			Very large	2014 questionnaire
U15	Unidentified sea turtle		Sighting	Alive	Sometime in 1994	Southern BC	Brooks Peninsula—off Cape Cook	50.133	−127.936	Best guess				Large	Spaven et al. (2009, 2003); questionnaire
U16	Unidentified sea turtle		Sighting	Alive	Mid 1990s	Southern BC	Kyuquot Sound—5 NM offshore at “Strawberry Patch” towards Esperanza Point	49.867	−127.267	Approximate					Spaven et al. (2009); hotline/outreach
U17	Unidentified sea turtle		Sighting	Alive	Aug 1998	Central BC	Darby Channel—near Lone Island	51.511	−127.742	Approximate	15.03		~45		Spaven et al. (2009, 2003); uestionnaire
U18	Unidentified sea turtle		Sighting	Alive	Late Aug 1998	Southern BC	Cape Cook—50 NM NW of	50.043	−129.250	Best guess					Spaven et al. (2009, 2003); questionnaire
U19	Unidentified sea turtle		sighting	Alive	Sometime in 1999	Southern BC	Nootka Sound	49.500	−126.600	Best guess					Spaven et al. (2009); hotline/outreach
U20	Unidentified sea turtle		Sighting	Alive	Jul 2000	Southern BC	Bajo Reef, Nootka Sound	49.583	−126.800	Approximate	14.32				Spaven et al. (2009); hotline/outreach
U21	Unidentified sea turtle		Sighting	Alive	Aug 2000	Southern BC	Nootka Sound—off Yuquot Point	49.595	−126.610	Best guess					Spaven et al. (2009, 2003); questionnaire
U22	Unidentified sea turtle		Sighting	Alive	Sep 2000	Northern BC	Hecate Strait—near weather buoy	53.625	−131.093	Approximate	12.77				Hotline/outreach
U23	Unidentified sea turtle		Mortality	Dead, unknown condition	Feb 2002	Southern BC	Tofino area	49.133	−125.917	Best guess				Not collected or examined	Spaven et al. (2009, 2003); questionnaire
U24	Unidentified sea turtle		Mortality	Dead, skeletal remains	2 Feb 2004	Northern BC	Rose Harbour	52.160	−131.066	Best guess				Not collected or examined	Spaven et al. (2009); hotline/outreach
U25	Unidentified sea turtle		Sighting	Alive	3 Jun 2007	Central BC	Addenbroke Point Lightstation—0.8 NM W of	51.603	−127.868	Approximate	11.8				Spaven et al. (2009); hotline/outreach
U26	Unidentified sea turtle		Sighting	Alive	19 Aug 2007	Southern BC	Barkley Sound—49 NM off	48.298	−126.322	Best guess					Spaven et al. (2009); hotline/outreach
U27	Unidentified sea turtle		Sighting	Alive	Sometime in 2009	Southern BC	Vancouver Island— offshore	CBD	CBD	Uncertain					2014 questionnaire
U28	Unidentified sea turtle		Sighting	Alive	23 May 2014	Southern BC	Strait of Georgia—W of Stradiotti Reef, S of Savary Island	49.919	−124.844	Approximate	12.84				Hotline/outreach
U29	Unidentified sea turtle		Sighting	Alive	4 Jul 2016	Southern BC	Halfmoon Bay	49.506	−123.926	Approximate	15.41			Creamy underbelly	Hotline/outreach
U30	Unidentified sea turtle		Sighting	alive	Apr 13 2020	Southern BC	Port McNeill	50.767	−127.335	Exact	8.69			Larger head than that of a pinniped	Hotline/outreach
U31	Unidentified sea turtle		Sighting	Alive	Almost every Aug	Northern BC	Cape St James	51.910	−131.030	Best guess				Observed at least one free swimming turtle almost annually in August (unknown year range) at this general location	2003 questionnaire
U32	Unidentified sea turtle		Sighting	Alive	Almost every Aug	Southern BC	Vancouver Island—off W side at 500 fathom edge	CBD	CBD	Uncertain				Observed one free swimming turtle almost every August (unknown year range) at this general location	Spaven et al. (2009, 2003); questionnaire
U33	Unidentified sea turtle		Sighting	Alive	Aug of unknown year	Southern BC	Sea Otter Cove	50.665	−128.370	Approximate					Spaven et al. (2009, 2003); questionnaire
U34	Unidentified sea turtle		sighting	Alive	Unknown date	Southern BC	Vancouver Island—W	CBD	CBD	Uncertain				Observed at least one live free turtle annually (unknown year range) at this general location	Spaven et al. (2009, 2003); questionnaire
U35	Unidentified sea turtle		Sighting	Alive	Unknown date	Northern BC	Haida Gwaii—E side	53.200	−131.400	Best guess					Spaven et al. (2009, 2003); questionnaire

### 3.1 Spatial Distribution

**FIGURE 2:**
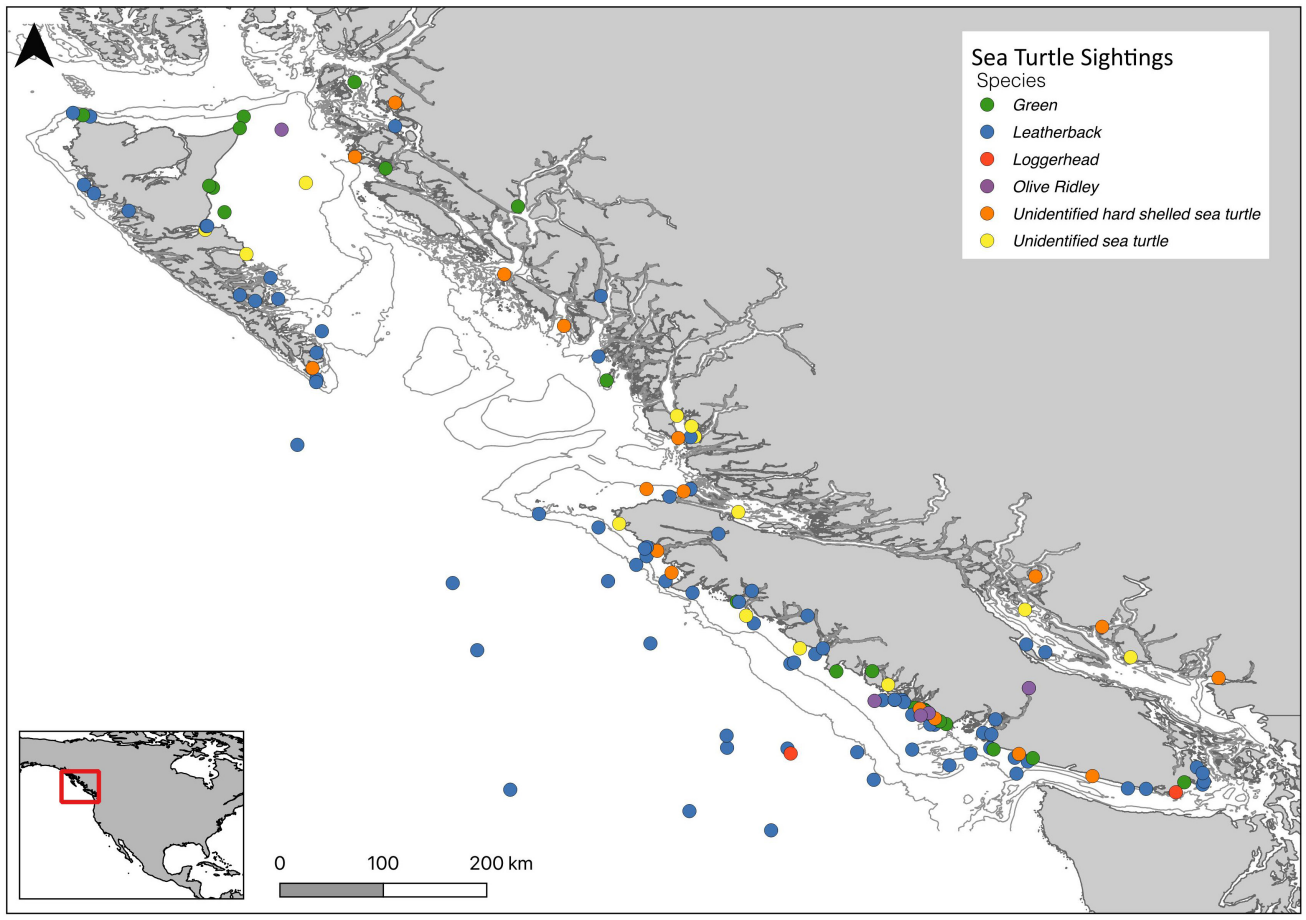
Distribution of all sea turtle records in BC waters with exact and approximate (within 5 km accuracy) locations (*n* = 140). Records include leatherback (*n* = 77, blue), green (*n* = 25, green), loggerhead (*n* = 3, red), olive ridley (*n* = 5, purple), unidentified hard‐shelled sea turtles (*n* = 17, orange), and unidentified sea turtles (*n* = 13, yellow). Bathymetry lines depict 100 and 200 m isobaths.

Sea turtles were sighted in all regions of coastal BC with 70% (*n* = 174) seen in southern waters, surrounding Vancouver Island. A total of 140 records were mapped (Figure 2). There were 102 records of free‐swimming turtles with known coordinates: 82% occurred in neritic waters on the continental shelf, 10% over the continental slope, and 8% offshore, often near seamounts. Since 2000, one third of live‐stranded or dead sea turtles have been found within and adjacent to Pacific Rim National Park Reserve (PRNPR), on the west coast of Vancouver Island (*n* = 10, including 5 greens, 3 olive ridleys, 1 leatherback, 1 unidentified hard‐shelled sea turtle). The most northerly free‐swimming sea turtle was a green in the fall of 1981 in Chatham Sound near the Alaskan border, reported by Hodge and Wing (2000) (G5).

### 3.2 Temporal Distribution

**FIGURE 3:**
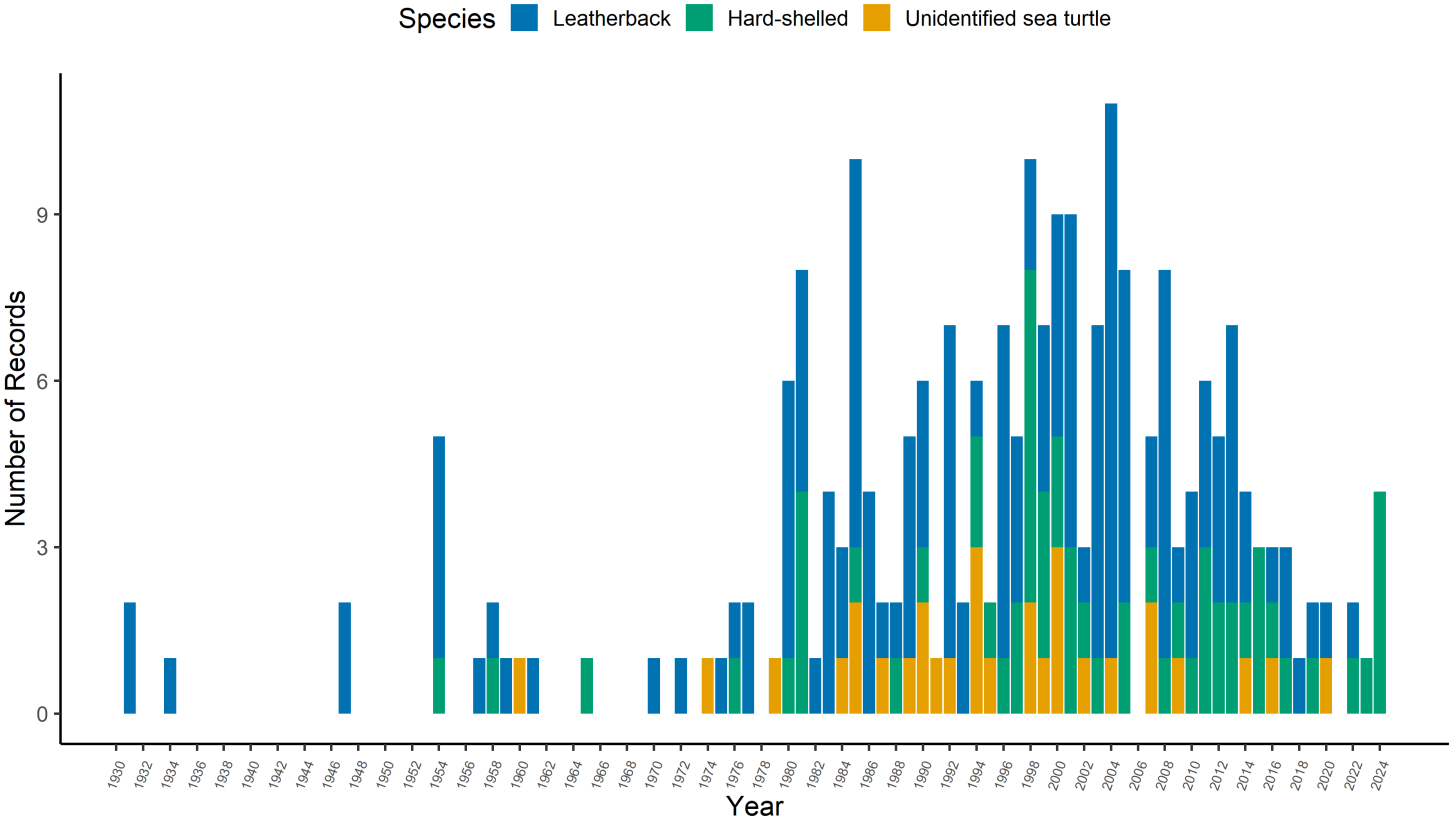
Annual distribution of sea turtles sighted in Canadian Pacific waters from 1931 to 2024 (*n* = 207 turtles), including leatherbacks (blue), hard‐shelled sea turtles (green), and unidentified sea turtles (yellow).

**FIGURE 4:**
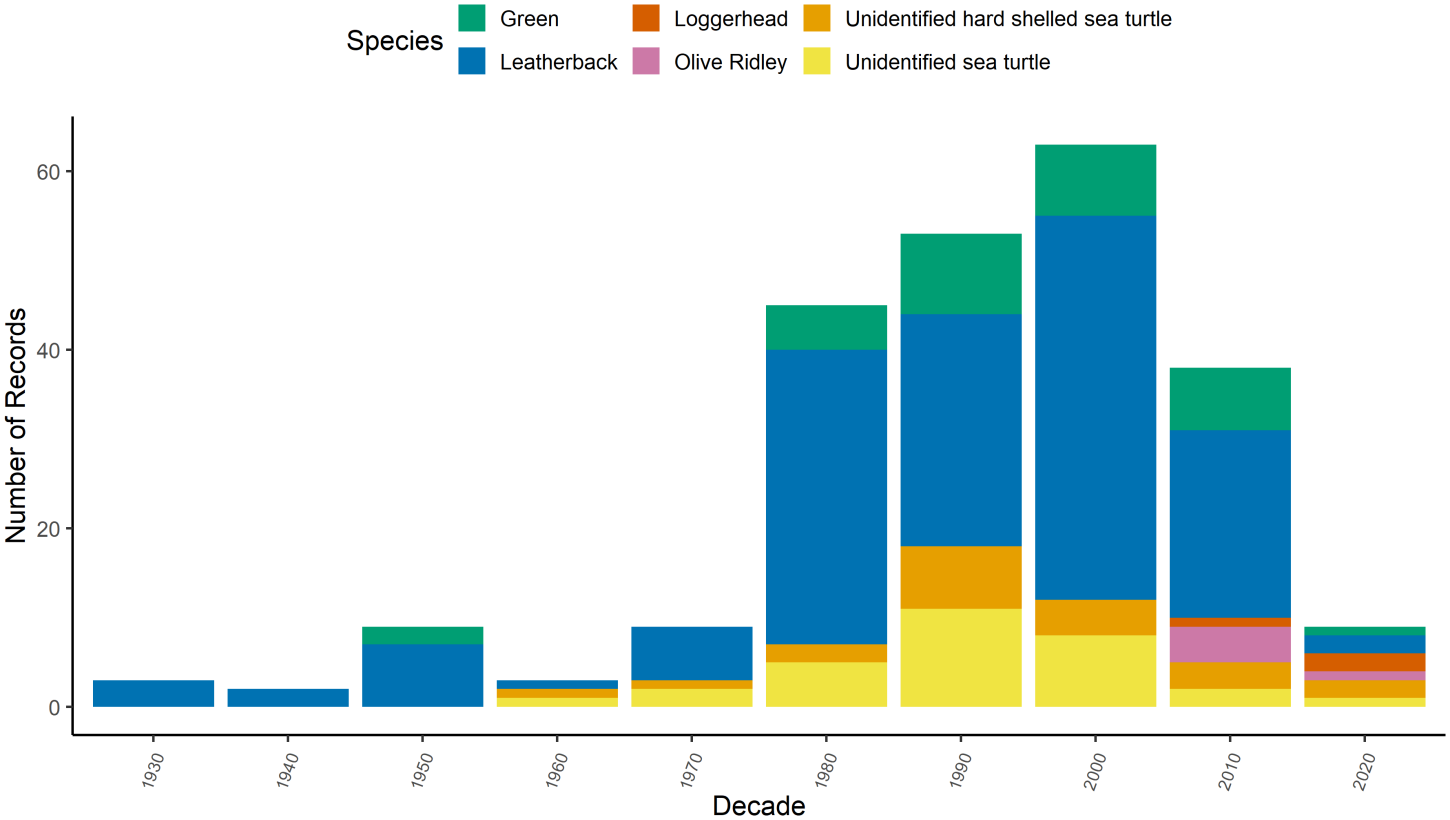
Number of sea turtles sighted in Canadian Pacific waters by decade (*n* = 238 turtles) including leatherbacks (blue), green (green), loggerhead (red), olive ridley (purple), unidentified hard‐shelled sea turtles (orange), and unidentified sea turtles (yellow).

**FIGURE 5:**
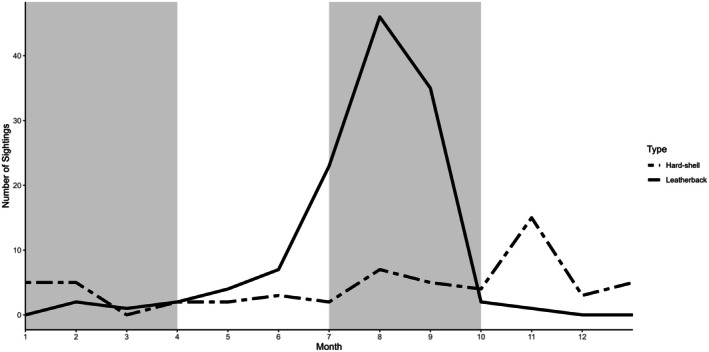
Seasonal occurrence of sea turtles (*n* = 179) in Canadian Pacific waters, depicted by month (*x* axis) and season (gray shading), including leatherback turtles (*n* = 125, solid line), and hard‐shelled sea turtles (combined green, loggerhead, olive ridley, unidentified hard‐shelled sea turtles (*n* = 54, dashed line)).

Sea turtles have been reported in 56 of the last 93 years (*n* = 203 turtles with year‐specific details). The most hard‐shelled sea turtles were seen in 1981 and 1998 (*n* = 6, 5 respectively; Figure 3) with the diversity of hard‐shelled species peaking in the 2010s (Figure 4). The highest number of leatherbacks were reported in 2004, 2005, and 2008 (*n* = 10, 7, 7 respectively; Figure 3). Sea turtles have been recorded in all months, most commonly in summer (67%, *n* = 138 of 207 with seasonal details; Figure 5): August (31%), September (23%) and July (15%). While summer accounted for 75% of all leatherback reports, fall was the most prominent season for hard‐shelled sea turtles (39%), mostly greens. Only hard‐shelled species have been observed in December and January. During a 2‐week period in late 2011, three sea turtles (one subadult female olive ridley (OR1) and two adult male greens (G26, G27)) stranded on a beach in PRNPR. Though presumed alive at the time of collection, none survived. Two of three loggerheads were reported in February (LH1, LH2).

### 3.3 Age Class and Size

Only 13 records included known sex and/or age class: nine males and three females; eight adults, four subadults, and a single juvenile. All but one male was an adult and all three females were subadults. Measured or estimated SCL was available for 87 records. All leatherbacks (*n* = 55) were over 1 m in length and 54% were over 2 m. All hard‐shelled sea turtles (*n* = 30) were under 1.5 m in length, and 70% were 50–100 cm in length. The 18 records with a measured curved or straight carapace length are included in Table 3. The smallest examined turtle was a juvenile green of unknown sex found dead in Haida Gwaii in November 2014, weighing 3.4 kg, with an SCL of 32 cm (G30).

**TABLE 3:** Measured morphometrics of sea turtles found in British Columbia from 1931 to 2024 (*n* = 18), including species, date, sex, age class, weights, and carapace lengths and widths when recorded.

Record #	Species	Date	Sex	Age class	Straight carapace length (cm)	Curved carapace length (cm)	Straight carapace width (cm)	Weight (kg)
LB1	Leatherback	Aug 1931	NA	NA	290	NA	300	635
LB2	Leatherback	Aug 1931	NA	NA	215	NA	NA	~660
LB3	Leatherback	Summer 1934	NA	NA	137	NA	320	545
LB10	Leatherback	Jul 1957	NA	NA	180	NA	86	296
G1	Green	Dec 1954	NA	NA	47	NA	NA	14.5
G17	Green	Jan 2000	NA	NA	87	NA	NA	NA
G19	Green	Nov 2001	NA	NA	78.7	NA	NA	NA
G20	Green	Dec 2001	Male	Adult	68.7	NA	NA	40.3
G21	Green	Jan 2002	Male	Adult	69.9	NA	NA	45.8
G22	Green	Aug 2005	Female	Sub adult	84	NA	61	NA
G23	Green	Jun 2007	NA	NA	57	NA	NA	NA
G27	Green	Dec 2011	Male	Adult	102	109	73	~40
G29	Green	Feb 2013	NA	NA	57	NA	45	NA
G30	Green	Nov 2014	NA	Juvenile	32	NA	26	3.4
G31	Green	Jan 2016	Male	Adult	67	71	52	NA
OR1	Olive ridley	Nov 2011	Female	Sub adult	70	75	NA	45
OR4	Olive ridley	Sep 2019	Male	Adult	64	66	59	26.9
LH2	Loggerhead	Feb 2024	Female	Sub adult	63	67.5	52	38

Abbreviation: NA, not assessed.

### 3.4 Mortality and Nonlethal Interactions

Almost 91% of leatherbacks were observed alive (*n* = 137 of 150), compared to 58% of hard‐shelled sea turtles (*n* = 36 of 62). Of the five reported olive ridleys, three were alive: one died enroute to rehabilitation, one was free swimming with an unknown fate, and one survived with rehabilitation after being found near Port Alberni in 2019 (OR3). There are three records of loggerheads: one free swimming (LH1), one found alive and collected for rehabilitation (LH2), and one deceased (LH3). In total, seven sea turtles were collected for rehabilitation care at VAMMR live stranding program (*n* = 4 green, 1 loggerhead, 2 olive ridley); four have survived and three have been released near San Diego, USA (*n* = 1 olive ridley (OR4), 1 green (G31), 1 loggerhead (LH2)).

Of the 47 sea turtles observed dead or that died during a response, 10 were collected (*n* = 6 green, 3 olive ridley, 1 leatherback) and underwent postmortem examinations. Unfortunately, the samples and results of the only necropsy conducted on a leatherback (May 1997, Kyuquot (LB71)) have not been located. The primary diagnoses for the remaining nine turtles were trauma to the carapace and/or plastron, multisystemic intravascular trematodes, fungal pneumonia, septicemia, cold stress, superficial algal colonization of the carapace and/or plastron, and emaciation (Table 4). One olive ridley had multiple longitudinal and oblique superficial incisions suspect of a propeller strike (OR1), and one green turtle had a defect on the caudal limit of the carapace suggestive of a shark attack (G20). Based on spatiotemporal stranding and pathology, cold stress was probable in four turtle deaths, and possible in two deaths. Hard plastic fragments were recovered from the stomach of one olive ridley (OR1), along with evidence of multifocal bleeding of the stomach serosa, but were not associated with the cause of death.

**TABLE 4:** Postmortem findings and cause of death determined by examination of sea turtles (*n* = 9) found in British Columbia. A frequency table of primary diagnoses is provided in Appendix C.

Record#	Species	Date	Status at time of observation	Pathology findings
G20	Green	Dec 2001	Dead, moderate decomposition	Trauma to the posterior margin of carapace, wounds suggestive of shark attack. Secondary sepsis, and presumptive cold stress
G21	Green	Jan 2002	Dead, advanced decomposition	Could not be determined due to post mortem change. Multisystemic intravascular and gastrointestinal trematode infection, liver hemosiderosis
G26	Green	Nov 2011	Alive, died	Probable cold‐stun, fungal pneumonia, multisystemic intravascular trematodes, and bacterial endocarditis, focal superficial defect of the caudal limit of the carapace (trauma)
OR1	Olive ridley	Nov 2011	Alive, died	Acute traumatic perforation of the left lateral aspect of the carapace with resolving superficial linear injuries to carapace and plastron, muscular hemorrhage with pneumocoelom, hemocoelom, and probable cold‐stun with inappetence. Note: plastic ingestion with serosal hemorrhage of the stomach (*not COD)
G27	Green	Dec 2011	Alive, died	Cold‐stun, multisystemic intravascular trematode ova with occasional granuloma formation, within normal limits
G28	Green	Feb 2012	Dead, moderate decomposition	Carapace, caudal margin, traumatic triangular defect with fragmented margins and secondary septicemia. Multisystemic intravascular trematodiasis
OR2	Olive ridley	Oct 2013	Dead, moderate decomposition	Generalized emaciation with superficial fungal and algal colonization of the carapace suggest possible cold‐stun, lethargy and generalized debilitation
G30	Green	Nov 2014	Dead, moderate decomposition	Emaciation with possible cold‐stun, autolyzed
OR3	Olive ridley	Feb 2015	Dead, moderate decomposition	Mycotic pneumonia with liver hemosiderosis algal colonization and overgrowth of the plastron suggestive of debilitation, lack of mobility, or lethargy

There are 16 human interactions noted in the dataset, and 75% of those were entanglements in fishing gear (*n* = 10 leatherbacks, 1 unidentified hard‐shelled sea turtle, 1 unidentified sea turtle; Table 5). Gillnet fishing was responsible for 58% (*n* = 7) of entanglement events and all turtles were released alive. Three leatherbacks were found entangled in seine nets and were released alive. One leatherback was found with its flipper caught on a trolling stabilizer line and was released alive (LB45). One leatherback was found dead, entangled in crab gear near Haida Gwaii (LB59). No fishing interactions have been reported since 1992. The remaining incidents include three leatherbacks shot in the 1930s (LB1‐LB3), and one report of a vessel collision with a leatherback while trolling at slow speeds (LB78)—no injuries were observed to the turtle.

**TABLE 5:** Reported sea turtle entanglement events with fishery gear type (*n* = 12) in British Columbia waters.

Record #	Species	Date	Gear type	Location	Lethal?
LB10	Leatherback	Jul 1957	Gillnet	Barkley Sound, SW Vancouver Island	Yes
U1	Unidentified hard‐shelled sea turtle	Jun/Jul 1958–1963	Gillnet	Rivers Inlet, central coast	No
UHS2	Unidentified sea turtle	1976 or 1977	Gillnet	Fitz Hugh Sound, central coast	No
LB21	Leatherback	Jul/Aug 1980	Seine net	Cape Mark, central coast	No
LB27	Leatherback	Aug 1981	Gillnet	Skidegate Inlet, E Haida Gwaii	No
LB44	Leatherback	Jun/July around 1986	Gillnet	Skeena River, North coast	No
LB45	Leatherback	Aug 1986	Trolling stabilizer	Nootka Sound, W Vancouver Island	No
LB49	Leatherback	Sep 1989	Gillnet	Carmanah Point, SW Vancouver Island	No
LB52	Leatherback	Around 1989	Gillnet	Nitnat, SW Vancouver Island	No
LB59	Leatherback	Aug 1992	Crab gear	Cape St James, S Haida Gwaii	Yes
LB146	Leatherback	Unknown	Seine net	Hippa Island, NW Haida Gwaii	No
LB148	Leatherback	Unknown	Seine net	Langara Island, N Haida Gwaii	No

### 3.5 Sea Surface Temperature

Estimated monthly SST readings were obtained for 83 records of free swimming sea turtles with exact or approximate coordinates, where the SST information was available through the HadISST dataset. The temperature ranged from 8.7°C to 16.7°C. All records from the summer months (July–September) were associated with an SST of 12.3°C or warmer and the average summer SST was 14.6°C. Live hard‐shelled sea turtles (*n* = 19) were seen in waters ranging from 9.2°C to 15.8°C. Of the leatherbacks with associated SST data (*n* = 56) all but three were in the summer months, and 88% occurred in temperatures greater than 12°C, averaging 14.5°C.

## 4 Discussion

It is unsurprising that on average less than three turtles are reported in Pacific Canadian waters each year, given the inconspicuous nature of sea turtles, the lack of dedicated survey effort, and being on the edge of the species ranges. While the leatherback is the most frequently sighted sea turtle in BC waters, it is still considered a rare occurrence (Hodge and Wing 2001). Greens are the second most sighted sea turtle in BC, followed by olive ridley, and loggerhead. Nonetheless, all three hard‐shelled species should be considered a casual occurrence in BC (Hodge and Wing 2001). It is likely that loggerhead and olive ridley sea turtle numbers are underrepresented, as 24% of sighted turtles remain unidentified, and may have been misidentified for the more commonly known green turtle, especially where photos were unavailable.

Most sightings occurred opportunistically, and many factors likely influenced the frequency of sightings. Compiled records show a gradual increase in sightings starting in the 1970s to a peak in the mid 2000s. This peak is reflective of an increase in sea turtle awareness efforts starting in the early 2000s, while the gradual increase corresponds with the career length of fishers who responded to the 2003 and 2014 questionnaires. However, reasons for the spike in hard‐shelled species reports in 1981 and 1998 are less obvious. The drop in leatherback sightings over the last 15 years could reflect the continued decline of the Western Pacific population (Wallace et al. 2025; Mazaris et al. 2017). Benson et al. (2020) note that leatherback abundance off California has declined by 5.6% annually without any significant changes in ocean conditions or prey availability. Systematic monitoring is required to confirm occurrence and distribution trends and to identify any potential foraging areas for sea turtles in BC (Gregr et al. 2014). Unfortunately, no dedicated sea turtle survey efforts have occurred for almost 20 years, and multispecies survey efforts have not yielded sufficient numbers of sightings for analysis.

Though sea turtle sightings occurred along the entire coast of BC, 82% were on the continental shelf and 70% occurred in the southern part of the province, overlapping spatially with a large proportion of boating and fishing activity. Southern BC is also closest to the core turtle range for all four populations (Wallace et al. 2023), so more sightings to the south are expected.

Many leatherback sightings occurred in coastal regions of Vancouver Island, and over offshore gullies and seamounts, aligning with favorable foraging conditions for gelatinous prey. Jellies aggregate and are retained in areas of upwelling shadows (Benson et al. 2003; Benson et al. 2007). Southwestern BC features considerable summer upwelling of nutrient‐rich waters due to the interactions of the Fraser River freshwater outflow, southwest summer winds and coastal currents like the nearshore northward coastal current, the southward shelf‐break current, the Juan de Fuca Eddy at the Canada–US border, and the southward flowing California current further offshore (Okey and Dallimore 2011). Based on modeled areas of high forage suitability and known occurrences, important foraging habitat for leatherbacks in the Canadian Pacific has been identified as the entire continental shelf of BC, excluding the mainland inlets, river deltas and portions of the Strait of Georgia (Fisheries and Oceans Canada 2014). The oceanographic features off southwestern Vancouver Island are also likely to have contributed to the rash of cold‐stunned hard‐shelled sea turtle strandings in and around Pacific Rim National Park Reserve.

Although sea turtle sightings were recorded year‐round, occurrences in summer months were most frequent, especially of leatherbacks. Hard‐shelled sea turtle sightings were more evenly distributed seasonally. As hard‐shelled sea turtles do not normally occur in BC waters their presence is likely accidental or vagrant, and these turtles are often either comatose, or dead, suggesting passive transportation with currents. While most sea turtles were reported alive, over half the hard‐shelled sea turtles were reported dead compared to a handful of leatherbacks, likely due to lower cold tolerances. Cold‐stunning was the leading cause of injury or death for necropsied hard‐shelled sea turtles in BC. Fortunately, two free swimming turtles collected for rehabilitation (G22 and OR4) were found in 14°C—15°C waters, about ~4°C–5°C warmer than the 10°C threshold below which cold‐stunning occurs (Schwartz 1978).

Studies suggest sea turtle presence is increasing at higher latitudes due to warming sea temperatures (Chaloupka et al. 2008; Avens and Dell'Amico 2018). These trends are not evident in BC data aside from a spike in hard‐shelled turtle records during the 1998 El Niño, one loggerhead record in 2015 (LH1), and two in early 2024 (LH2 and LH3) during another strong El Niño. Several fishers anecdotally linked sightings to El Niño events (DFO unpublished data). With El Niño events projected to become more frequent (Wang et al. 2017), especially in areas where ocean dynamics may shift rapidly, mortality events may also increase (Osland et al. 2021). However, El Niño events suppress ocean upwelling (Okey and Dallimore 2011), and thus may reduce prey availability for leatherbacks during these warmer periods. In contrast, La Niña events may enhance upwelling and improve habitat conditions. Following the 2015–2016 marine heat wave, Santidrián Tomillo et al. (2020) found sea turtle nesting populations were negatively impacted by warming conditions, projecting declines for Eastern Pacific leatherbacks and olive ridleys, and likely affecting other populations. Although no clear patterns of range expansion or sightings fluctuations due to water warming and cooling events seem to be occurring in BC waters to date, ongoing monitoring is essential to detect shifts in population dynamics, which may heighten vulnerability to fisheries and climate impacts.

The 12 sea turtle entanglement reports in BC represent a minimum number at best. Wallace et al. (2010) note that both bycatch and entanglements of sea turtles go largely unreported, and therefore the number of turtles entangled in BC is likely much higher than is known. While Canada's Fisheries Act requires immediate reporting of any accidental contact between a marine mammal and a vessel or fishing gear, sea turtle interaction reporting is not similarly required. Globally, bycatch and mortality rates of sea turtles are highest in gillnet and trawl fisheries, particularly in small‐scale fisheries (Lewison and Crowder 2007; Alfaro‐Shigueto et al. 2011; Eguchi et al. 2017). In BC, no sea turtle entanglements have been reported since 1992, but historically involved gillnets, followed by seine nets. The use of gillnets in commercial BC salmon fisheries has since declined (Bertram 2023; Fisheries and Oceans Canada 2022b; Wood 2008); however current First Nations food, social and ceremonial (FSC) fishing in BC still includes gillnet use. While this fishery poses some risk to sea turtles, the Pacific Salmon Strategy Initiative management measures support a transition to more selective harvesting methods (e.g., seining, trolling, and fish wheels) that may help avoid future sea turtle bycatch and mortality. Continued efforts should also be made to reduce the presence of “ghost” or derelict gear in BC waters (Fisheries and Oceans Canada 2022a) to ensure safe passage for foraging turtles.

Threat assessments of sea turtles in migratory and foraging habitats elsewhere have highlighted a need for monitoring of vessel strike (Hazel and Gyuris 2006; Welsh and Witherington 2023), plastic ingestion (Schuyler et al. 2016; Wilcox et al. 2018), and harmful algal blooms (Perrault et al. 2017, 2020). Although no trends have yet come to light in BC, evidence of blunt force trauma indicative of vessel strike was found in several dead turtles, and plastic debris was found in the stomach of a dead olive ridley. Future strandings should be assessed for evidence of these types of human interactions.

## 5 Conclusions and Future Directions

This paper establishes the current and complete dataset of sea turtle occurrence in Canadian Pacific waters. The relatively low number of confirmed reports underscores the importance of documenting rare sea turtle sightings in detail and highlights the necessity to sustain and promote these efforts. With potential range expansion due to climate change, and continued population level declines, it is prudent to enhance monitoring strategies to establish a better understanding of sea turtle presence in this region. It would also be valuable to require mandatory sea turtle bycatch reporting in all fisheries, to better understand the impacts of fishing practices, particularly for the critically endangered leatherback. Work is ongoing to collect and analyze jellyfish distribution and density data off western Vancouver Island to help identify areas of concentration for leatherback prey species. With a better understanding of their foraging habitat, effort‐based research can help refine the boundaries of critical habitat necessary to protect and recover leatherbacks.

## Author Contributions

**Lisa Spaven:** conceptualization (lead), data curation (lead), formal analysis (equal), investigation (equal), methodology (lead), project administration (lead), resources (equal), validation (lead), visualization (equal), writing – original draft (lead), writing – review and editing (lead). **Amy Migneault:** conceptualization (supporting), formal analysis (equal), resources (equal), visualization (equal), writing – original draft (supporting), writing – review and editing (supporting). **Karina Dracott:** conceptualization (supporting), formal analysis (equal), resources (equal), visualization (equal), writing – original draft (supporting), writing – review and editing (supporting). **Caitlin Birdsall:** data curation (supporting), funding acquisition (equal), investigation (equal), methodology (supporting), resources (equal), validation (supporting), writing – review and editing (supporting). **Tessa Danelesko:** data curation (supporting), funding acquisition (equal), investigation (equal), methodology (supporting), resources (equal), validation (supporting), writing – review and editing (supporting). **Stephen Raverty:** data curation (supporting), funding acquisition (equal), investigation (equal), resources (equal), validation (supporting), writing – review and editing (supporting). **Martin Haulena:** data curation (supporting), funding acquisition (equal), investigation (equal), resources (equal), validation (supporting), writing – review and editing (supporting). **John K. B. Ford:** funding acquisition (equal), methodology (supporting), resources (equal), writing – review and editing (supporting).

## Conflicts of Interest

The authors declare no conflicts of interest.

## Data Availability

All data are available in Tables 1 through 5. Explanations for data classification, filtering and summation are described in the Methods section. Note that Tables 2 through 5 and Appendix C are linked by a unique record identifier.

## Appendix B Publicly Available Awareness and Outreach Resources Used in Gathering Sea Turtle Occurrence Reports

1. Sea turtle and ID Guide (OceanWise Sightings Network) https://oceanorg.blob.core.windows.net/oceanorg/2024/01/OWSN_IDGuides_2024.pdf.
2. They're Out There You Know: Leatherbacks in BC! animation (Marine Education & Research Society) https://www.youtube.com/watch?v=sej8s6WQn2c&ntb=1&msockid=13c175251be711f0814c0a08dfbdd03c.
3. WhaleReport App (OceanWise) https://ocean.org/action/send‐a‐sighting‐save‐a‐whale/.

## Appendix C Pathology Findings Frequency Table From Post‐Mortem Examinations of Sea Turtles

**Table:**

Record #	Species	Date	Trauma	Trematodiasis	Fungal pneumonia	Septicemia	Cold stress	Algal colonization	Comment
G20	Green	Dec 2001	Moderate, posterior margin or carapace, possible shark attack	NA	NA	Moderate	Probable	NA	
G21	Green	Jan 2002	NA	Moderate, multisystemic	NA	NA	NA	NA	
G26	Green	Nov 2011	Mild. Focal defect posterior limit carapace	Moderate, multisystemic	moderate	NA	Probable	NA	
OR1	Olive ridley	Nov 2011	Marked, penetrating trauma with superficial lacerations	NA	NA	NA	Probable	NA	Inappetence with plastic ingestion with hemorrhage of gastric serosa
G27	Green	Dec 2011	NA	Moderate, multisystemic	NA	NA	Probable	NA	
G28	Green	Feb 2012	Moderate, triangular defect posterior margin of carapace	Moderate, multisystemic	NA	NA	NA	NA	
OR2	Olive ridley	Oct 2013	NA	NA	NA	NA	Possible	Moderate	Emaciation
G30	Green	Nov 2014	NA	NA	NA	NA	Possible	NA	Emaciation
OR3	Olive ridley	Feb 2015	NA	NA	moderate	NA	NA	Moderate	
		Totals	4	4	2	1	6	2	3
		Sub Totals	Mild = 1, Moderate = 2, Marked = 1	Moderate = 4	Moderate = 2	Moderate = 1	Possible = 2, Probable = 4	Moderate = 2	Emaciation = 2, Plastic ingestion = 1
